# Anti-diabetic and anti-inflammatory bioactive hits from *Coriaria intermedia* Matsum. stem and *Dracontomelon dao* (Blanco) Merr. & Rolfe bark through bioassay-guided fractionation and liquid chromatography-tandem mass spectrometry

**DOI:** 10.3389/fphar.2024.1349725

**Published:** 2024-03-08

**Authors:** Mavis Colleen Porciuncula Fabian, Rezzaira Marie Neduelan Astorga, Arnelson Arwin Gray Atis, Luis Agustin Elido Pilapil, Christine Chichioco Hernandez

**Affiliations:** Bioorganic and Natural Products Laboratory, Institute of Chemistry, University of the Philippines Diliman, Quezon City, Philippines

**Keywords:** anti-diabetic, anti-inflammation, enzyme inhibition, dereplication, LC-MS/MS, α-glucosidase, 15-lipoxygenase-1, terrestrial plants

## Abstract

Women have been found to be at a higher risk of morbidity and mortality from type 2 diabetes mellitus (T2DM) and asthma. α-Glucosidase inhibitors have been used to treat T2DM, and arachidonic acid 15-lipoxygenase (ALOX15) inhibitors have been suggested to be used as treatments for asthma and T2DM. Compounds that inhibit both enzymes may be studied as potential treatments for people with both T2DM and asthma. This study aimed to determine potential anti-diabetic and anti-inflammatory bioactive hits from *Coriaria intermedia* Matsum. stem and *Dracontomelon dao* (Blanco) Merr. & Rolfe bark. A bioassay-guided fractionation framework was used to generate bioactive fractions from *C. intermedia* stem and *D. dao* bark. Subsequently, dereplication through ultra-high performance liquid chromatography-tandem mass spectrometry (UHPLC-MS/MS) and database searching was performed to putatively identify the components of one bioactive fraction from each plant. Seven compounds were putatively identified from the *C. intermedia* stem active fraction, and six of these compounds were putatively identified from this plant for the first time. Nine compounds were putatively identified from the *D. dao* bark active fraction, and seven of these compounds were putatively identified from this plant for the first time. One putative compound from the *C. intermedia* stem active fraction (corilagin) has been previously reported to have inhibitory activity against both α-glucosidase and 15-lipoxygenase-1. It is suggested that further studies on the potential of corilagin as an anti-diabetic and anti-inflammatory treatment should be pursued based on its several beneficial pharmacological activities and its low reported toxicity.

## Introduction

Diabetes is a chronic disease that is characterized by abnormally high plasma glucose levels. This is caused by the body’s inability to produce insulin or use insulin properly (Kaul et al., 2023). Type II diabetes (T2DM) is the most common form of diabetes, characterized by the body’s inability to synthesize and secrete insulin, which prevents the intake of glucose and leads to the subsequent rise of blood glucose levels. On the other hand, asthma is a chronic disease of the lungs affecting people from all age groups, caused by inflammation and the tightening of the muscle around the airways (World Health Organization, 2023). It is characterized by airway obstruction and hypersensitivity, thickening of the airway wall, and increased hypersecretion of mucus (Liu et al., 2023). Combined, diabetes and asthma affect hundreds of millions of people around the world annually (Chowdhury et al., 2021; Sun et al., 2021; Diabetes, 2023).

Women are disproportionately affected by T2DM and asthma compared to men (Klein et al., 1999; Adair et al., 2018; Naeem and Silveyra, 2019; Pennington et al., 2019). There is also a shift observed in asthma prevalence and mortality rates in males and females over time, which suggests a role played by hormonal changes during puberty as well as the interaction of various socioeconomic and health factors (Naeem and Silveyra, 2019; Chowdhury et al., 2021). Therefore, it is crucial to develop new therapeutic agents for the treatment and/or management of T2DM and asthma, especially for women.

Lifestyle modifications are prescribed and several classes of oral medications are used to manage and treat T2DM (Padhi et al., 2020; Reed et al., 2021). One such class of medications used to treat T2DM are called α-glucosidase inhibitors. On the other hand, asthma is most commonly managed using a combination of multiple medications (García-Menaya et al., 2019). In a recent review (Xu et al., 2021), it was mentioned that human arachidonic acid 15-lipoxygenase (ALOX15) inhibitors have the potential to be used as treatments for airway inflammatory diseases, including asthma. ALOX15 and its metabolites have been suggested to also play a role in T2DM and the complications that arise from it (Singh and Rao, 2019; He et al., 2023), implying that ALOX15 inhibitors may be explored as a possible treatment for T2DM and/or its complications. Several compounds and plant extracts that inhibit α-glucosidase or ALOX15 have already been studied (Kumar et al., 2011; Sadeghian and Jabbari, 2015). However, there might be value in exploring compounds/extracts that inhibit both enzymes as possible sources of new treatments for T2DM and asthma.

The immense biodiversity of terrestrial plants makes them a rich source of chemically diverse secondary metabolites with promising biological activities (Atanasov et al., 2015). Given the rich biological niche of the Philippine archipelago, its flora represents an untapped chemical space with the potential to yield novel drug leads. Several Philippine plants, such as *tuway-tuway* (*Bidens pilosa* L.), *takip-kohol* (*Hydrocotyle asiatica* L.), and *gumamela* leaves (*Hibiscus rosa-sinensis* L.), have been used in traditional medicine to treat diabetes and inflammation. The medicinal properties of these plants are likely attributed to a variety of phytochemical constituents, including alkaloids, glycosides, and triterpenoids (Clemen-Pascual et al., 2022).

The current study is part of a government-funded research program called the *Tuklas Lunas* Research & Development program, previously known as the Discovery and Development of Health Products program (Philippine Council for Health Research and Development, Department of Science and Technology, 2024). The program aims to produce medicines from Philippine biodiversity by pursuing two tracks of drug development in parallel: (1) “the development of standardized herbal drugs”, and (2) “the identification and characterization of high-value purified active compounds from marine and terrestrial sources for specific therapeutic indications”. The initial phase of the program involved pre-screening 2,400 plant extracts from different regions of the Philippines against different enzyme-based bioassays. Cytotoxicity testing on the active extracts was then conducted and only the noncytotoxic extracts were further evaluated.

As part of our ongoing efforts to identify bioactive hits from Philippine plant extracts, we focused on exploring the bioactivity of two Philippine plants: *Coriaria intermedia* Matsum., and *Dracontomelon dao* (Blanco) Merr. and Rolfe. These two plants were chosen to be pursued for further studies because of their significant bioactivity in preliminary studies. The crude methanolic extracts of both *C. intermedia* stem and *D. dao* bark exhibited high levels of activity against α-glucosidase and 15-lipoxygenase-1. The crude extracts from both plants were also shown to be noncytotoxic in preliminary cytotoxicity assays and they were chosen as priority extracts based on the results of the cell-based orthogonal assays conducted by one of the program collaborators.

*Coriaria intermedia* Matsum.*,* commonly known as *beket* in Tagalog, is a tree native to the Cordillera Central Range of the northern Philippines (Guron and Napaldet, 2020). Plants from this genus are found in a variety of geographic locations, including Western North America and East Asia (Good, 1930). In Taiwan, *C. intermedia* has been traditionally used for treatment of gastrointestinal disturbance, rheumatism, and uterus cancer (Chang et al., 1996). The leaves of the plant were found to contain a variety of natural products, including 20-epibryonolic acid, coriamyrtin, ursolic acid, 3,3′-dimethyl ether, naringenin, β-tutin and phytosterols. Methanolic extracts from the leaves and flowers of *C. intermedia* exhibited significant antimicrobial activity (San Luis et al., 2014).

*Dracontomelon dao* (Blanco) Merr. and Rolfe, locally known as *paldao* or *maliyan*, is a deciduous tree under the family Anacardiaceae, and is widely distributed in the Philippines, Thailand, Myanmar, Cambodia, and southern China (Dapar, 2021). It holds significant ethnopharmacological value due to its diverse medicinal applications. In China, its bark is traditionally used to treat skin ulcers and other infectious diseases (Li et al., 2017). Similarly, in the Philippines, its bark is widely used as a traditional medicine to address sore throats, toothaches, and even as a relief for women who underwent labor (Peña et al., 2019). *D. dao* leaves were previously found to contain phytyl fatty acid esters, long-chain fatty alcohols, and long-chain hydrocarbons. Various secondary metabolites have also been isolated from its leaves, including anacardic acid, phytol, and β-sitosterol (Ragasa et al., 2016). In addition, its twigs were found to contain linoleic acid, cardanols, stigmasterol, anacardic acid, and monoacylglycerol, further exemplifying its rich phytochemical diversity (Ragasa et al., 2017). The solvent partition fractions from the methanolic extracts of the leaves, stem, root, and bark of *D. dao* exhibited a significant level of broad-spectrum antimicrobial activity (Khan and Omoloso, 2002). The ethyl acetate partition fraction of the ethanolic extract of *D. dao* leaves was also found to exhibit wound healing effects on bacterially infected wounds in rats (Wen et al., 2022).

Our group’s research involves the discovery of bioactive compounds from terrestrial plants using a bioassay-guided fractionation framework. Bioassay or bioactivity-guided fractionation is a common strategy used in natural products research (Hubert, Nuzillard, and Renault, 2015). However, bioassay-guided fractionation is often time- and resource-intensive. To reduce the amount of time required to discover new bioactive natural products, it is suggested that a dereplication procedure be involved in the workflow.

Dereplication refers to the rapid identification of secondary metabolites that have previously been identified (Hubert, Nuzillard, and Renault, 2015). There have been significant improvements in metabolite profiling methods due to the introduction of ultra-high-performance liquid chromatography (UHPLC) and the development of benchtop high-resolution mass spectrometry detectors (Allard et al., 2016) which are able to produce high quality tandem MS data. Coupled with molecular networking platforms such as the Global Natural Products Social Molecular Networking (GNPS), which can efficiently identify secondary metabolites using tandem MS data (Yang et al., 2013), the dereplication of natural products can be expedited.

There have been previous similar studies that have utilized bioassay-guided fractionation and LC-MS/MS analysis coupled with database or library searching to study natural products. In one study, 280 fractioned samples from 35 marine fungal strains from China were evaluated for their acetylcholinesterase (AChE) inhibitory activity and antioxidant activity using a thin layer chromatography array autography-based AChE inhibition assay and 2,2-diphenyl-1-picrylhydrazyl (DPPH) free radical scavenging assay (Nie et al., 2020). Their cytotoxicity was also determined using the *Artemia* larval lethality assay. The most bioactive and least cytotoxic fraction was analyzed via bioactivity-coupled LC-MS/MS, and the LC-MS/MS data was also uploaded to GNPS for molecular networking. Twelve compounds were identified to exhibit either AChE inhibitory activity or DPPH radical scavenging activity, with 7 of these compounds being putatively identified, and five compounds were suggested to be new compounds. In another study, the cytotoxic effects of semi-purified fractions from the ethyl acetate extract of *Annona muricata* L. leaves were evaluated against A549 cancer cells using *in vitro* MTS cytotoxicity and scratch/wound healing assays (Salac et al., 2022). Two subfractions (F15-16C and F15-16D) were identified to show the highest anticancer activity, and the fraction F15-F16 was further analyzed using LC-MS/MS, with the obtained data being analyzed using several metabolomics tools, including GNPS. Feature-based molecular networking using GNPS produced 28 hits or putative compounds from the F15-16 fraction.

This study aims to determine potential anti-diabetic and anti-inflammatory bioactive hits from *C. intermedia* stem and *D. dao* bark through a framework utilizing bioassay-guided fractionation, coupled with dereplication using UHPLC-MS/MS and database searching. To the best of our knowledge, this will be the first study which will use this framework for studying bioactive fractions from *C. intermedia* stem and *D. dao* bark.

## Materials and methods

### General

Distilled technical grade methanol, hexane, ethyl acetate, and ethanol were used to produce the crude plant extracts and the preliminary fractions by vacuum liquid chromatography (VLC). Analytical reagent (AR) grade hexane (JT Baker), ethyl acetate (JT Baker), ethanol (Scharlau, JT Baker), methanol (JT Baker), acetonitrile (RCI Labscan, JT Baker), DMSO (RCI Labscan), and ultrapure water (18.2 MΩ) (OmniaTap, stakpure GmbH) were used for the further fractionation of the VLC fractions via various separation methods, and as needed for the bioassays.

### Preparation of plant materials and extraction

*C. intermedia* stems (accession number UPB-0090) were collected from Halsema Highway, Atok, Benguet. The stems were washed and air-dried for 5–7 days, and were then homogenized in a stainless-steel pulverizer.

*D. dao* bark (accession number PUH 13423) were collected from the University of the Philippines Diliman Campus, Quezon City. The bark was washed, dried at 40°C in an oven for at least 3 days, and ground in a stainless-steel pulverizer.

The extraction and solvent partitioning procedures used were optimized in the laboratory and adapted from previous protocols (Punzalan and Villaseñor, 2019a). Approximately 1.9 kg of dried *C. intermedia* stems and approximately 1.4 kg of dried *D. dao* bark were soaked in distilled methanol, separately, for at least 3 days. The methanol soakings for both plants were then separately filtered and concentrated using a rotary evaporator (BÜCHI Rotavapor R-100). The water bath was set to 40°C and the chiller (BÜCHI Recirculating Chiller F-105) was at 2.5°C. The extraction process was repeated at least 7 times for each plant.

Solvent partitioning was performed on the methanolic extracts of *C. intermedia* stem and *D. dao* bark. Approximately 25–26 g of methanolic extract was dissolved in 500 mL ultrapure water and then partitioned with 500 mL distilled hexane. The hexane extract was separated and concentrated using a rotary evaporator, and the addition of distilled hexane to the aqueous mixture was repeated at least 6 times. To extract the slightly polar components, 500 mL distilled ethyl acetate was shaken with the remaining aqueous portion from the initial aqueous mixture. Similarly, extraction of the ethyl acetate extract was performed at least 6 times, and the extract was subsequently concentrated. The remaining aqueous mixture was then filtered to remove insoluble particles and stored at −20°C. A total of 53.30 g and 76.91 g of methanolic extract were partitioned for *C. intermedia* stem and *D. dao* bark, respectively.

### Bioassay-guided fractionation

The fractionation procedures used were optimized in the laboratory and adapted from previous protocols (Abaya and Villaseñor, 2019; Punzalan and Villaseñor, 2019b). The ethyl acetate extracts of *C. intermedia* stem and *D. dao* bark were subjected to VLC. The samples were prepared by mixing the extracts with silica gel 60G for thin layer chromatography (Merck) in a 1:1 sample to silica weight ratio. Silica gel 60G was also used as packing material for the stationary phase, using a 1:30 sample to silica weight ratio. Dry silica was loaded and packed with the assistance of a vacuum pump onto a glass column with a radius of 4.25 cm and a medium porosity glass frit. Prior to loading the sample, the stationary phase was equilibrated using distilled hexane. Stepwise gradient elution was employed for both samples using a solvent system of distilled hexane and ethyl acetate, starting with 100% hexane, then changing the concentration of each solvent in 10% increments, until 100% ethyl acetate was reached. Fractions were collected by volume for each solvent system and concentrated using the rotary evaporator.

For *C. intermedia* stem, 5.492 g of ethyl acetate extract was used for VLC with a packed column height of 6.5 cm. The total volume used per solvent system is 500 mL. To recover highly polar compounds, stepwise gradient elution using a solvent system of distilled ethyl acetate and distilled ethanol was performed, starting from 100% distilled ethyl acetate, until 100% distilled ethanol, in 25% increments. The collected fractions were concentrated.

*D. dao* bark was subjected to VLC twice (5.038 g and 4.681 g), both times with a column height of 5.5 cm and a mobile phase of 250 mL per solvent system. Compounds with higher polarity were recovered by using 50% distilled ethyl acetate:50% distilled ethanol mixture, followed by 100% distilled ethanol. The collected fractions from both runs were pooled and concentrated.

Size-exclusion chromatography (SEC) was performed to fractionate the 13th VLC fraction (which eluted from the 50% ethyl acetate:50% ethanol solvent system) of the *C. intermedia* stem ethyl acetate extract. The packing material used for the stationary phase was Sephadex LH-20 (Sigma-Aldrich, LH20100), made to swell overnight in AR grade methanol. The stationary phase was loaded onto a glass column with a radius of 1 cm and a medium porosity glass frit. The resulting packed stationary phase had a height of 58.5 cm. The sample was prepared by dissolving 105 mg of the 13th VLC fraction in 1.5 mL AR grade methanol, which was then centrifuged prior to loading. AR grade methanol was used as the mobile phase, and the fractions were collected by band and volume. Based on the obtained UHPLC-MS profiles of the SEC fractions, fractions 24 to 31 were pooled together and concentrated using a rotary evaporator. Fractionation of the 13th VLC fraction using SEC was repeated three times. The total weight of sample used for SEC was 315 mg.

The pooled *C. intermedia* stem SEC fraction was then fractionated further through solid phase extraction (SPE). The fraction was dissolved in 100% ultrapure water. The SPE cartridge (Bond Elut C18 500 mg/3 mL; Agilent) was activated using AR grade acetonitrile, then equilibrated with ultrapure water. The sample was loaded onto the cartridge, then washed with ultrapure water. Stepwise gradient elution was performed, starting with 100% ultrapure water until 100% AR grade acetonitrile, in 10% increments. All the fractions were collected per solvent system and concentrated using a refrigerated centrifugal vacuum concentrator (Labconco). The first fraction is henceforth referred to as **CINS**.

Further fractionation of the 12th VLC fraction (eluted from 100% ethyl acetate) of *D. dao* bark ethyl acetate extract was done through gravity column chromatography (GCC). For the stationary phase, silica gel 60 (0.063–0.200 mm) for column chromatography (Merck) was used as packing material. A 1:100 sample to silica weight ratio was followed. The stationary phase was loaded onto a glass column with a radius of 1 cm, and a medium porosity glass frit. The solvent used for column equilibration and as the initial solvent was 30% hexane:70% ethyl acetate. The fraction (142.2 mg) was dissolved with 30% hexane:70% ethyl acetate and loaded onto the column through wet loading. Stepwise gradient elution and collection by band and volume were employed, which resulted in a total of 15 GCC fractions. Based on the profiles of the 15 fractions obtained using UHPLC-MS, similar fractions were pooled together. Pooled GCC fraction 4 (henceforth referred to as **DDAB**) was then subjected to UHPLC-MS/MS analysis.

All the extracts and fractions generated were subjected to α-glucosidase and 15-lipoxygenase-1 inhibition assays.

### α-glucosidase inhibition assay

The α-glucosidase inhibitory activities of the extracts and fractions were determined via a spectrophotometric enzyme assay based on the Tuklas Lunas Protocols for Drug Discovery and Development in the Philippines (Naing et al., 2019). The assay was based on the procedure of Shobana et al. (2009) with modifications. Modifications of the assay were established from optimization experiments such as determination of the optimal enzyme concentration, K_m_ and V_max_ determination, determination of the IC_50_ of the positive control, and solvent tolerance of the enzyme.

The reaction mixture contained phosphate buffer (50 mM NaH_2_PO_4_ (Loba Chemie Pvt. Ltd.)/Na_2_HPO_4_ (Loba Chemie Pvt. Ltd.) with 100 mM NaCl (Scharlau) buffer, pH = 6.8), α-glucosidase from *Saccharomyces cerevisiae* recombinant (EC 3.2.1.20; Sigma-Aldrich, G0660), sample/inhibitor, and p-nitrophenyl-α-D-glucopyranoside (p-NPG) (Merck). The enzyme inhibitory activity was tested by plating 10 µL of the sample (300 ppm), 216 µL of the phosphate buffer, and 24 µL of the α-glucosidase solution (120 mU/mL) into a 96-well quartz microplate (Hellma Analytics). After incubation at 37°C for 10 min, 50 µL of p-NPG (11.34 mM) was added to start the reaction. Using a microplate spectrophotometer (Thermo Scientific Multiskan GO) and the software SkanIt RE v7.0, the absorbance was monitored at 405 nm every 30 s for 30 min. The positive control used was acarbose (Sigma-Aldrich, A8980-1G, SLCF5122), and the negative control used was 5% DMSO in phosphate buffer.

The % inhibition was calculated using the following equation:

$$\%\;Inhibition\;per\;replicate=\frac{{Absorbance}_{uninhibited}\;-{\;Absorbance}_{inhibited}\;}{{Absorbance}_{uninhibited}}\times 100$$

Dose-response curves for CINS, DDAB and acarbose against the α-glucosidase assay were constructed, Supplementary Figures S13–S15 and the IC_50_ values were calculated. For the effective concentrations of CINS, DDAB and acarbose used in the determination of the IC_50_ values, refer to Supplementary Table S1.

### 15-Lipoxygenase-1 inhibition assay

A spectrophotometric assay for the determination of lipoxygenase inhibitory activity was employed following the Tuklas Lunas Protocols for Drug Discovery and Development in the Philippines (Allanigue and Hernandez, 2019). The assay was based on the procedures of Auerbach et al. (1992) and Axelrod et al. (1981) with modifications. Modifications of the assay were established from optimization experiments such as determination of the optimal enzyme concentration, K_m_ and V_max_ determination, determination of the IC_50_ of the positive control, and solvent tolerance of the enzyme.

The reaction mixture contained phosphate buffer (0.1 M KH_2_PO_4_ (Loba Chemie Pvt. Ltd.)/K_2_HPO_4_ (Loba Chemie Pvt. Ltd.), pH = 7.4), lipoxidase (15-lipoxygenase-1) from *Glycine max* (soybean) Type I-B (EC 1.13.11.12; Sigma-Aldrich L7395), sample/inhibitor, and linoleic acid (Sigma-Aldrich, L1376). The enzyme inhibitory activity was tested by plating 10 µL of the sample (1,000 ppm), 260 µL of the phosphate buffer, and 15 µL of the enzyme solution (1896 U/mL) into a 96-well quartz microplate (Hellma Analytics). After incubation at 25°C for 5 min, linoleic acid (1.6085 mM) was added to start the reaction. Using a microplate spectrophotometer (Thermo Scientific Multiskan GO) and the software SkanIt RE v7.0, the absorbance was monitored at 234 nm every 10 s for 5 min. The positive control used was nordihydroguaiaretic acid (NDGA) (Sigma-Aldrich, 74,540-1G, BCBX4406), and the negative control used was 10% DMSO in phosphate buffer.

The % inhibition was calculated using the following equation:

$$\%\;Inhibition\;per\;replicate=\frac{{Absorbance}_{uninhibited}\;-{\;Absorbance}_{inhibited}\;}{{Absorbance}_{uninhibited}}\times 100$$

Dose-response curves for CINS, DDAB and NDGA against the 15-lipoxygenase-1 assay were constructed, Supplementary Figures S16–S18 and the IC_50_ values were calculated. For the effective concentrations of CINS, DDAB and NDGA used in the determination of the IC_50_ values, refer to Supplementary Table S2.

For both the α-glucosidase and the 15-lipoxygenase-1 assays, extracts and fractions that exhibit ≥50% inhibition were considered to be active against the enzyme.

### Statistical analyses

The assays were performed with three trials, with 4 replicates per trial. The enzyme inhibitory activities are presented as average ± standard deviation. IBM SPSS Statistics 26 was used to conduct the statistical analysis of the assay data. One-sample Kolmogorov-Smirnov test was done to check the normality of the distribution of slopes for each replicate, followed by Levene’s test to check the equality of the variances from the different test groups. Brown-Forsythe and Welch tests were done afterward, since the variances were unequal (*p* < 0.05). The data was then analyzed using Tamhane T2 - One-way ANOVA with unequal variances to check if the samples are considered active. Grubbs’ test (Graphpad outlier calculator) was used to determine any outliers in the replicates. GraphPad Prism 9 was used to calculate the IC_50_ values. The results of the statistical analyses of the assay data can be found in Supplementary Table S3–8.

### Dereplication through Ultra-High-Performance Liquid Chromatography–Tandem Mass Spectrometry (UHPLC-MS/MS)

The UHPLC-MS/MS analysis and dereplication procedures were optimized in the laboratory and adapted from previous protocols (Junio et al., 2019).

Fractions were dissolved in LC-MS grade methanol (Merck) at a concentration of 500 μg/mL. The sample solutions were centrifuged, and the supernatants were transferred to LC-MS-certified vials (Waters). UHPLC-MS experiments were conducted on a Waters H-Class series system with a Xevo G2-XS quadrupole time of flight (QToF) mass spectrometer. A 0.3 mL/min flow rate was used with an Acquity UPLC HSS T3 1.8 µm (2.1 × 100 mm) column (Waters). The mobile phase was composed of varying ratios of LC-MS grade acetonitrile (Merck) and water (Merck), both with 0.1% formic acid. The composition of the acetonitrile used was as follows: 10% at 0.00–2.00 min, increasing from 10% to 100% at 2.00–8.00 min, 100% at 8.00–10.00 min, decreasing from 100% to 10% at 10.00–11.00 min, and 10% at 11.00–12.00 min. The injection volumes used for the samples were 1 µL for the positive mode and 3 µL for the negative mode.

Base peak intensity (BPI) chromatograms were obtained for 12 min per sample and spectra were acquired in ESI positive and negative modes within the range of 50–1,200 Da. ESI source parameters for the positive mode were set as follows: capillary voltage = 2.80 kV, sampling cone voltage = 40 V, source offset = 80 V, source temperature = 120°C, desolvation temperature = 450°C, cone gas = 50 L/h, and desolvation gas = 800 L/h. For the negative mode, ESI source parameters were set as follows: capillary voltage = 1.50 kV, sampling cone voltage = 30 V, source offset = 80 V, source temperature = 120°C, desolvation temperature = 500°C, cone gas = 50 L/h, and desolvation gas = 1000 L/h. Mass spectrometry data were collected using two modes: MS and data dependent acquisition (DDA). For the DDA mode, MS/MS acquisition was done on ions that exceeded an intensity threshold of 1.0 × 10^5^ and 1.0 × 10^4^ for the positive and negative modes, respectively. A maximum of 10 ions were selected for MS/MS acquisition from a 0.5-s MS scan. Three collision energy ramps (15–30 eV, 30–45 eV, and 45–60 eV) were set to acquire extensive data on the small molecules.

MassLynx v4.1 was used to view and obtain the BPI chromatograms of the fractions. Peak alignment and conversion of the RAW data file to mgf file were executed using MSDIAL, v4.9.221218 (Tsugawa et al., 2015). The data were then uploaded to the Global Natural Products Social Molecular Networking (GNPS) platform (Wang et al., 2016) using WinSCP v6.1.1.

The feature-based molecular networking (FBMN) module of the GNPS platform (Nothias et al., 2020) was used to match the data acquired using DDA mode with existing spectral databases to obtain the putative identities of the compounds found in the active fractions. The criteria for matching were also adapted from a previous study (Molino et al., 2021) and optimized in the laboratory. The parameters for the FBMN were set as follows: precursor ion mass tolerance = 0.02 Da and fragment ion mass tolerance = 0.50 Da, a maximum of 5.00 for the ppm error, a minimum cosine score of 0.70, and a minimum of 6 for the matched fragment ions. The mirror matches of the experimental spectra with the reference spectra were also considered as criteria for the determination of the putative hits.

## Results

### Bioassay-guided fractionation

The preliminary results of the bioassays done on *C. intermedia* stem and *D. dao* bark extracts and fractions can be found in Supplementary Figures S7-12. The methanolic extract of *C. intermedia* stem (12.20% yield) both exhibited α-glucosidase **(AGLUC)** (99.91% ± 0.04%) and 15-lipoxygenase-1 **(LOX)** (91.44% ± 0.11%) inhibitory activities. Solvent partitioning was performed on the methanolic extract to separate the nonpolar, slightly polar, and the polar extracts. *C. intermedia* stem ethyl acetate extract was fractionated using VLC because it exhibited higher inhibition against both AGLUC (99.85% ± 0.03%) and LOX (92.90% ± 0.28%). Moreover, the ethyl acetate extract also had a higher yield (20.63%) compared to the hexane extract (5.29%). A total of 15 fractions were produced from the VLC. Out of the 15 fractions, 5 fractions were active against AGLUC only, and 1 fraction was active against LOX only. Six fractions were found to be active against both AGLUC and LOX and the 13th VLC fraction was selected since it had the highest yield (26.56%).

SEC was used to further fractionate the 13th VLC fraction. From this, 31 fractions were generated through isocratic elution. Thirteen out of the 31 fractions exhibited AGLUC inhibitory activity only. Eight fractions (fractions 24–31) exhibited inhibitory activity against both AGLUC (96.57% ± 0.65% to 99.73% ± 0.11%) and LOX (48.26% ± 2.47% to 91.96% ± 0.53%). Upon UHPLC-MS profiling, it was observed that all eight fractions have almost the same chromatogram. Since all eight fractions contained all the major peaks, they were pooled together into 1 fraction. The pooled fraction was then fractionated using a C18 SPE cartridge, which generated 12 fractions. The first SPE fraction (**CINS**) was chosen for dereplication and IC_50_ screening since it had the highest yield among the 12 fractions.

The AGLUC and LOX inhibitory activities of *D. dao* bark methanolic extract (12.82% yield) were 99.24% ± 0.22% and 99.26% ± 1.16%, respectively. Similar to the *C. intermedia* stem ethyl acetate extract, the *D. dao* bark ethyl acetate extract was pursued for fractionation since it exhibited high inhibitory activities against AGLUC (97.02% ± 1.59%) and LOX (97.73% ± 1.12%). Moreover, the *D. dao* bark ethyl acetate extract also had a higher yield (18.14%) than the hexane extract (8.87%).

The ethyl acetate fraction was fractionated through VLC. Out of the 25 VLC fractions of *D. dao* bark ethyl acetate, 12 fractions were found to be active against AGLUC and LOX. Two fractions exhibited inhibition against AGLUC only. The 12th VLC fraction was prioritized due to its high AGLUC (99.90% ± 0.03%) and LOX (108.22% ± 1.03%) inhibitory activities.

The 12th VLC fraction was further fractionated through GCC. Out of the 15 GCC fractions, 11 fractions showed inhibition against both AGLUC and LOX. One fraction was active against AGLUC only, and 1 fraction was active against LOX only. Based on the BPI chromatograms of the fractions obtained through UHPLC-MS, GCC fractions 6–11 (pooled GCC fraction 4) were pooled together. The pooled GCC fraction 4 **(DDAB)** had the highest yield among all the pooled fractions. DDAB was also selected for dereplication and IC_50_ screening.

### Enzyme inhibitory assays

Table 1 summarizes the IC_50_ results from the enzyme assays that were done on the prioritized active fractions. The IC_50_ values reported here for each sample/control are the average of the three trials per sample/control.

**TABLE 1:** IC_50_ values from enzyme inhibition assays on CINS and DDAB.

Sample	IC_50_ (in µg/mL)	
	AGLUC	LOX
CINS	0.25 ± 0.06	2.57 ± 0.86
DDAB	0.25 ± 0.04	3.58 ± 0.96
Acarbose	129.52 ± 14.01	—
NDGA	—	0.64 ± 0.25

It can be observed that both CINS and DDAB exhibited markedly lower IC_50_ values as compared to acarbose in the AGLUC inhibition assay. On the other hand, both CINS and DDAB exhibited greater IC_50_ values compared to NDGA in the LOX inhibition assay. This implies that the compounds present in these fractions are more potent inhibitors of AGLUC compared to acarbose but are less potent inhibitors of LOX compared to NDGA.

### Dereplication

**FIGURE 1:**
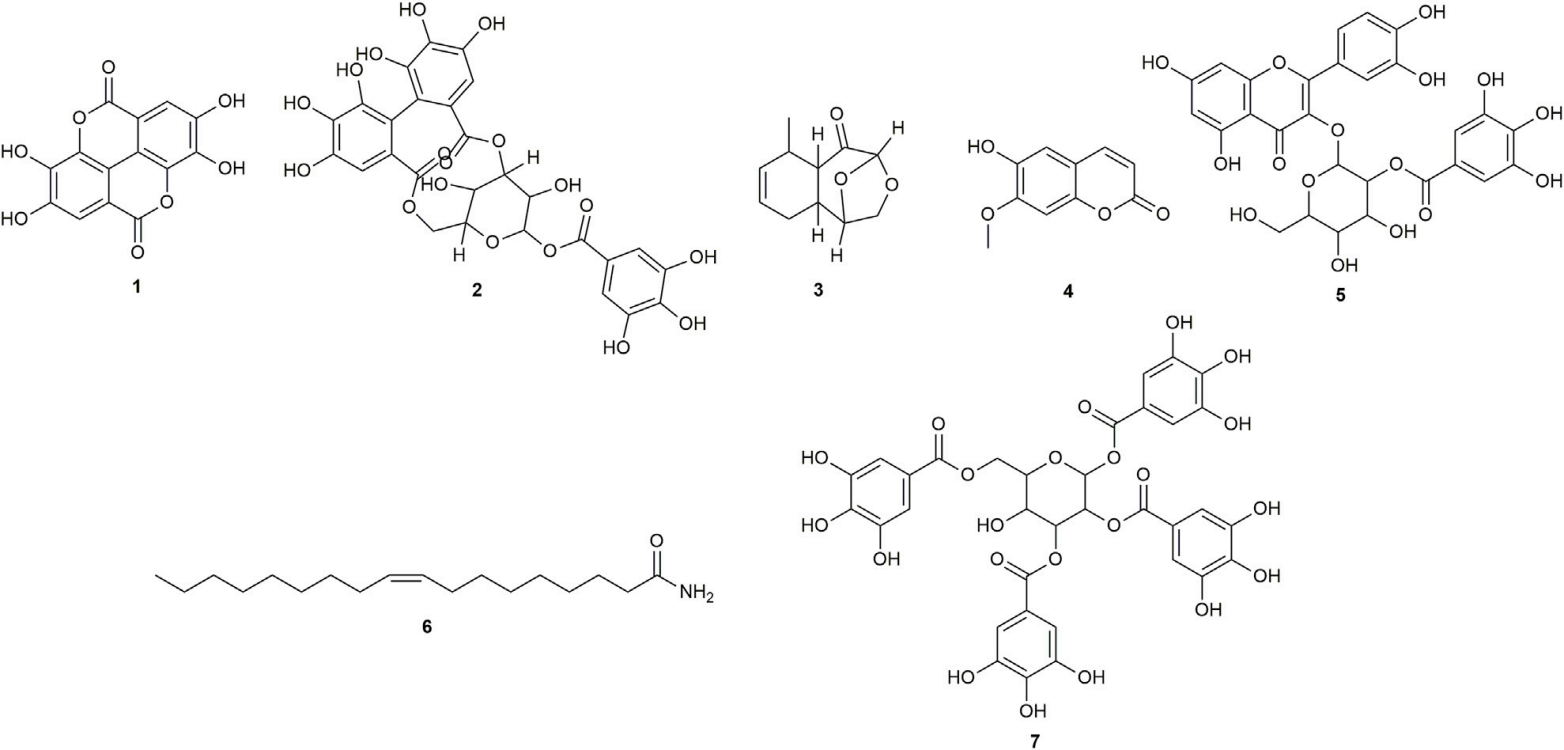
Chemical structures of compounds from CINS that were putatively identified by dereplication. Ellagic acid (**1**), corilagin (**2**), (1S,2S,6R,7R, 9R)-6-methyl-10,12-dioxatricyclo [7.2.1.0 < 2,7>]dodec-4-en-8-one (**3**), isoscopoletin (**4**), 2-O-galloylhyperin (**5**), oleamide (**6**) and 1,2,3,6-tetra-O-galloyl-beta-D-glucose (**7**).

**FIGURE 2:**
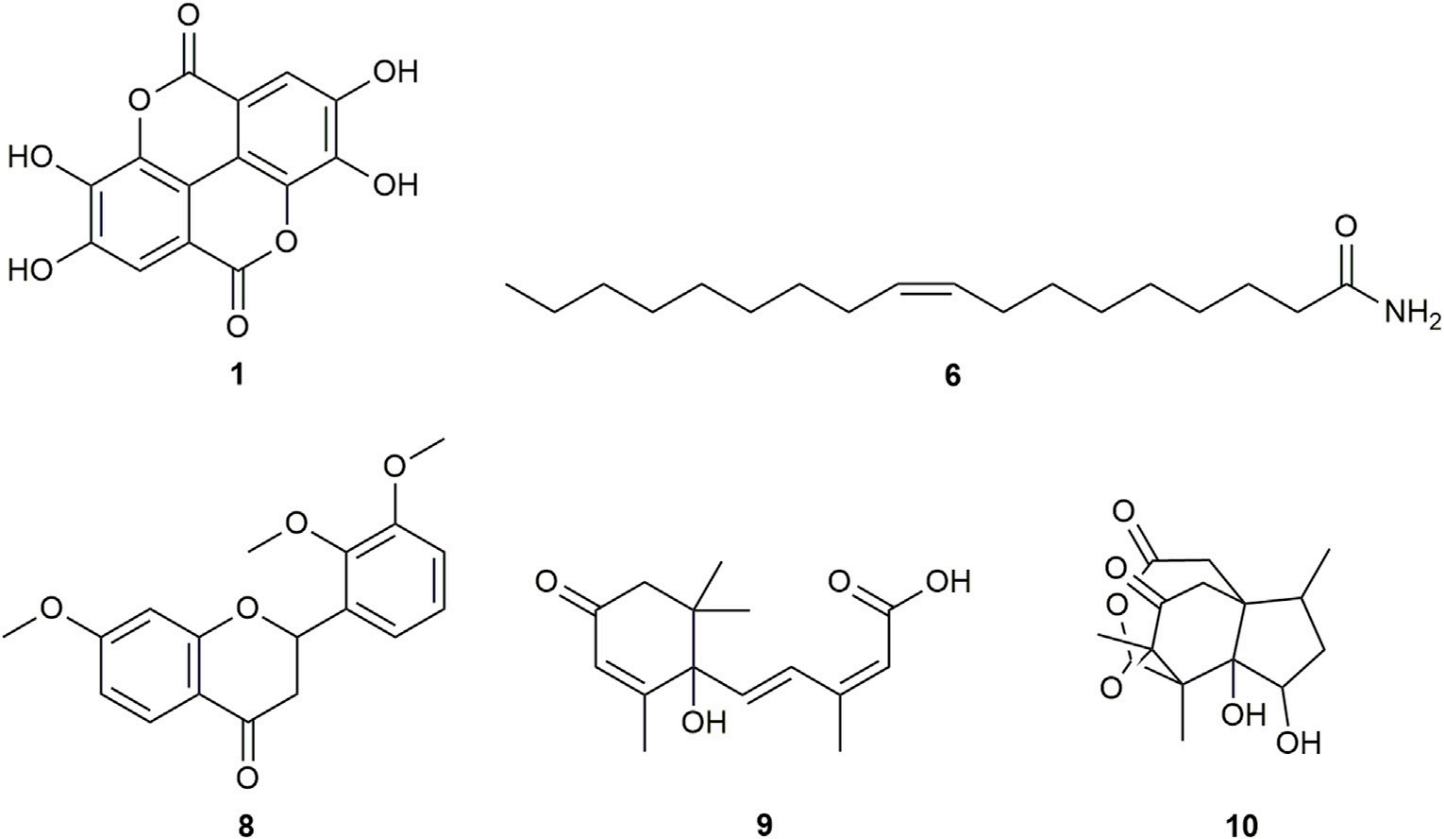
Chemical structures of compounds from DDAB that were putatively identified by dereplication. Ellagic acid (**1**), oleamide (**6**), 7,2′,3′-trimethoxyflavanone (**8**), abscisic acid (**9**), and pseudoanisatin (**10**).

Multiple compounds were putatively identified through the dereplication of CINS and DDAB. These compounds are summarized in Table 2; Figure 1 for CINS, and Table 3; Figure 2, 3 for DDAB.

**TABLE 2:** Putatively identified compounds from CINS using GNPS (GNPS^a^, PubChem^b^).

	Compound	Acquisition mode	Retention time (min)	Precursor Adduct	Experimental Mass	Monoisotopic Mass	ppm error	Cosine score
1	ellagic acid	positive	5.908	[M + H]^+^	303.0129	303.01^a^	9.60^a^	0.89
						303.0141^b^	−3.90^b^	
2	corilagin^c^	positive	5.096	[M + Na]^+^	657.0693	657.07^a^	−1.00^a^	0.87
						657.0704^b^	−1.59^b^	
		negative	4.932	[M-H]-	633.08362	633.067^a^	25.93^a^	0.81
						633.0728^b^	17.11^b^	
3	(1S,2S,6R,7R,9R)-6-methyl-10,12-dioxatricyclo [7.2.1.0 < 2,7>]dodec-4-en-8-one^c^	positive	9.059	[M + H]^+^	195.1018	195.102^a^	−1.23^a^	0.86
						195.1021^b^	−1.84^b^	
4	isoscopoletin^c^	positive	5.247	[M + Na]^+^	215.0331	215.032^a^	4.98^a^	0.77
						215.0320^b^	4.85^b^	
5	2-O-galloylhyperin^c^	positive	5.716	[M + H]^+^	617.1143	617.113^a^	2.04^a^	0.76
						617.1142^b^	4.86 × 10^−4b^	
6	oleamide^c^	positive	11.571	[M + H]^+^	282.2795	282.279^a^	1.91^a^	0.76
						282.2797^b^	−0.53^b^	
7	1,2,3,6-tetra-O-galloyl-beta-D-glucose^c^	negative	5.502	[M-H]-	787.0974	787.1^a^	−3.26^a^	0.75
						787.0994^b^	−2.53^b^	

^c^ First report of putative identification in *C. intermedia* stem.

**TABLE 3:** Putatively identified compounds from DDAB using GNPS (GNPS^a^, PubChem^b^).

	Compound	Acquisition mode	Retention time (min)	Precursor Adduct	Experimental Mass	Monoisotopic Mass	ppm error	Cosine score
1	ellagic acid	negative	5.708	[M-H]^-^	301.0005	300.999^a^	4.97^a^	0.82
						300.9984^b^	7.00^b^	
6	oleamide^c^	positive	11.596	[M + H]	282.2795	282.279^a^	1.91^a^	0.81
						282.2797^b^	−0.53^b^	
8	7,2′,3′-trimethoxyflavanone^c^	positive	9.091	[M + Na]^+^	337.1041	337.105^a^	−2.79^a^	0.91
						337.1052^b^	−3.36^b^	
9	abscisic acid	positive	6.845	[M + H-H_2_O]	247.1331	247.133^a^	0.40^a^	0.89
						247.1334^b^	−1.29^b^	
10	pseudoanisatin^c^	positive	6.57	[M + Na]^+^	321.1318	321.131^a^	2.40^a^	0.83
						321.1314^b^	1.13^b^	
11	1,3,6-tri-O-galloyl-beta-D-glucose^c^	positive	5.418	[M + Na]^+^	659.0850	659.09^a^	−7.56^a^	0.78
						659.0860^b^	−1.54^b^	
		negative	1.042	[M-H]^-^	635.0891	635.089^a^	0.19^a^	0.77
						635.0884^b^	1.06^b^	
12	1,2,4,6-tetra-o-galloyl-beta-D-glucose^c^	positive	5.627	[M + H]^+^	789.1142	789.115^a^	−1.01^a^	0.70
						789.1150^b^	1.07^b^	
13	pyrocatechuic acid^c^	negative	2.952	[M-H]^-^	153.0184	153.019^a^	−3.89^a^	0.86
						153.0188^b^	2.57^b^	
14	pyrogallol^c^	negative	1.56	[M-H]^-^	125.0233	125.024^a^	−8.18^a^	0.76
						125.0239^b^	4.71^b^	

^c^ First report of putative identification in *D. dao* bark.

**FIGURE 3:**
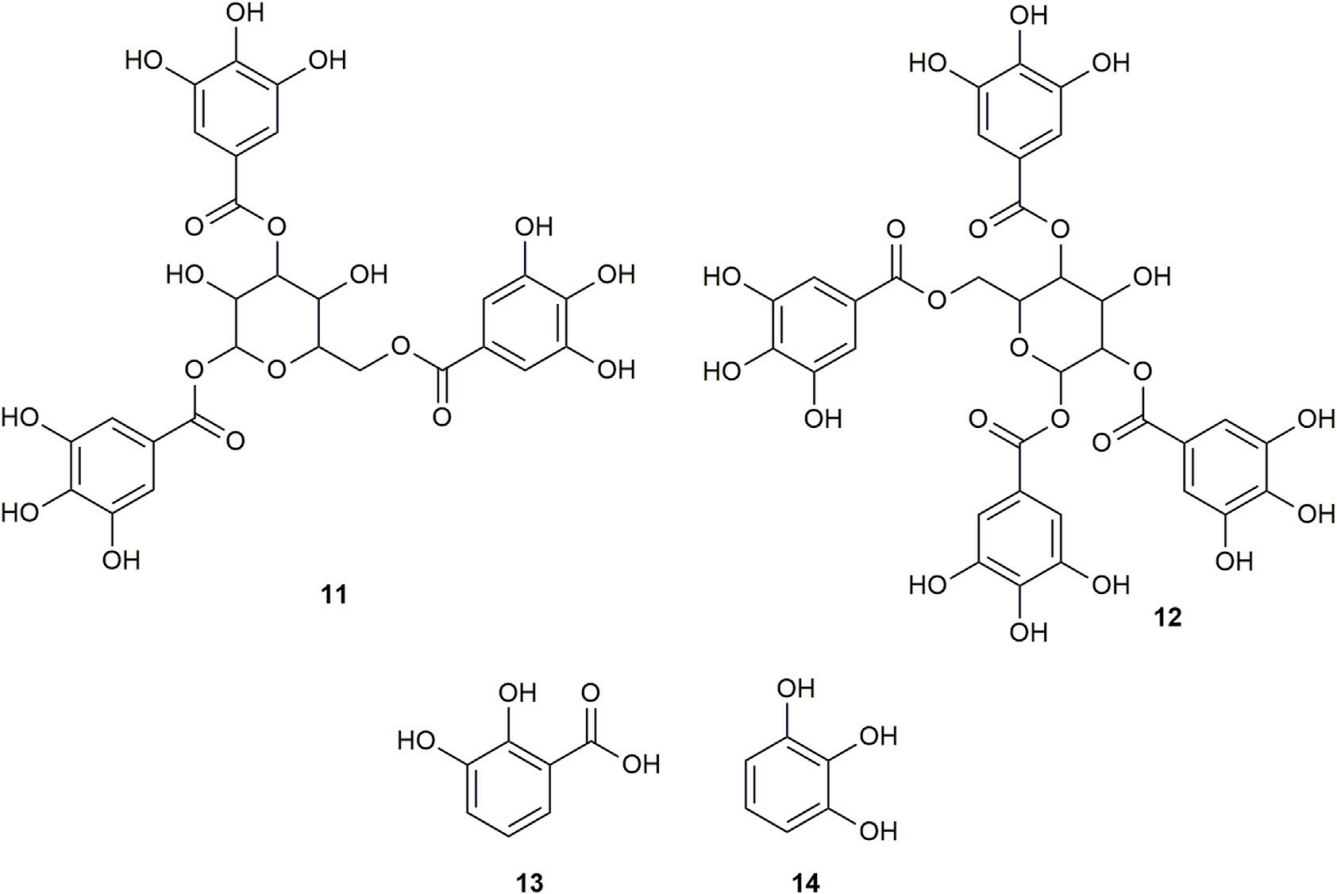
Chemical structures of compounds from DDAB that were putatively identified by dereplication. 1,3,6-tri-O-galloyl-beta-D-glucose (**11**), and 1,2,4,6-tetra-O-galloyl-beta-D-glucose (**12**), pyrocatechuic acid (**13**) and pyrogallol (**14**).

Based on the dereplication results, a literature search was conducted on the putative hits for both CINS and DDAB to see whether there have been previous studies on their antidiabetic and/or anti-inflammatory activities, which might explain the fractions’ bioactivities that were observed in the enzyme inhibition assays.

Ellagic acid (compound **1**) is a phenolic compound that was previously identified in the hexane fraction from the methanolic extract of *C. intermedia* leaves and roots (Chang et al., 1996), and can be found in numerous plants such as the leaves of *Tectaria subtriphylla* (Hook. and Arn.) Copel. (Feng-Lin and Jhy-Yih, 1993), the leaves and bark of *Acer negundo* L. (Saleh et al., 1969), and the bark of *Mallotus japonicus* (L.f.) Müll.Arg. (Yoshida et al., 1982). It also has anti-diabetic and anti-inflammatory properties (Ríos et al., 2018). More specifically, it was found to exhibit inhibitory activity against AGLUC with an IC_50_ value of 2.18 μg/mL (You et al., 2012). Its anti-inflammatory mechanisms include the inhibition of lipopolysaccharide (LPS)-induced nitric oxide (NO), prostaglandin E2 (PGE2), and interleukin-6 (IL-6) production (BenSaad et al., 2017). Compound **1** was putatively identified from both CINS and DDAB fractions.

Corilagin (compound **2**) is a hydrolysable tannin that was previously isolated from the shoot of *Geranium thunbergii* Siebold & Zucc. (Okuda et al., 1975), the roots of *Phyllanthus emblica* L. (Zhang et al., 2000) and the leaves of *Phyllanthus niruri* L. (Colombo et al., 2009). In addition, it was also isolated from the leaves of *Terminalia macroptera* Guill. and Perr. and exhibited AGLUC and LOX inhibitory activities with IC_50_ values of 2.58 ± 0.08 µM and 41 ± 4 μM, respectively (Pham et al., 2014).

For (1S,2S,6R,7R, 9R)-6-methyl-10,12-dioxatricyclo [7.2.1.0 < 2,7>]dodec-4-en-8-one (compound **3**), no previous studies have been conducted which quantified its antidiabetic properties using the AGLUC inhibition assay, and its anti-inflammatory activity using the LOX inhibition assay. Furthermore, this compound has not been previously identified from a plant source.

Isoscopoletin (compound **4**) is a hydroxycoumarin that has been isolated from various plants such as the aerial parts of *Tagetes lucida* Cav. (Céspedes et al., 2006), *Euphorbia hirta* L. (Wu et al., 2012) and from the stem wood of *Zanthoxylum integrifoliolum* (Merr.) Merr. (Chen et al., 2007). Furthermore, it also has antidiabetic activities that is exemplified by a variety of mechanisms such as the inhibition of advanced glycation end products formation (IC_50_ = 18.78 ± 0.50 µM) (Jung et al., 2012). Moreover, it also exhibited inhibitory activities towards α-amylase, maltase, and sucrase with % inhibition values of 0.2%, 32.2%, and 20.9%, respectively, at a concentration of 400 µM (Kato et al., 2008). Its anti-inflammatory properties can be attributed to its ability to inhibit LOX (IC_50_ = 15.1 µM) (Deng et al., 2007).

2-O-galloylhyperin (compound **5**) is a flavonoid glycoside that was isolated from the aerial parts of *Rubus amabilis* Focke (Chen et al., 2001), *Pyrola incarnata* (DC.) Fisch. ex Freyn (Yazaki et al., 1989), *Euphorbia lunulata* Bunge (Nishimura et al., 2005) and the leaves of *Eucalyptus globulus* Labill. (Park et al., 2023).

Oleamide (compound **6**) is a fatty acid amide that is found in numerous plants such as the stem of *Desmos cochinchinensis* Lour. Sun et al. (1995), the leaves of *Polygonum maritimum* L. (Rodrigues et al., 2017) and the leaves of *Lithocarpus polystachyus* (Wall. ex A. DC.) Rehder (Fang et al., 2022). It was reported with an IC_50_ value of 30.69 ± 1.47 μg/mL against AGLUC (Fang et al., 2022). Moreover, it demonstrated anti-inflammatory effects through various mechanisms such as the inhibition of LPS-induced nuclear factor kappa B (NF-κB) activation (Oh et al., 2010) and the suppression of p38, ERK, and PI 3-kinase/Akt and ROS accumulation (Moon et al., 2018). Compound 6 was putatively identified from both CINS and DDAB fractions.

1,2,3,6-tetra-O-galloyl-beta-D-glucose (compound **7**) is a tannin that was putatively identified in *Corchorus olitorius* L. leaf extract through UHPLC-MS (Alara et al., 2023). It was also isolated from the stem bark of *Bersama abyssinica* subsp. *abyssinica* and *Bersama abyssinica* subsp. *paullinioides* (Nyamboki et al., 2021) and the leaves and branches of *Phyllanthus emblica* L. Zhang et al. (2002).

7,2′,3′-trimethoxyflavanone (compound **8**) is a flavanone. Flavanones are responsible for the bitter taste of citrus peels and fruits, and they exhibit antioxidant, anti-inflammatory, blood lipid-lowering, and cholesterol-lowering properties (Panche et al., 2016).

Abscisic acid (compound **9**) is an isoprenoid phytohormone that is responsible for several regulatory plant functions such as growth. It can be found in numerous plants such as the leaves of *Cinnamomum subavenium* Miq. Kuo et al. (2008) and the epiphyte, *Cuscuta pentagona* Engelm (Kimura et al., 1982). Moreover, this compound can be a promising treatment for diabetes as it regulates glucose uptake *in vitro* and can stimulate insulin production in beta pancreatic cells (Xu et al., 2018). In addition, it was found that abscisic acid intake reduces neuroinflammation (Jeon et al., 2020) and colon inflammation (Guri et al., 2011).

Pseudoanisatin (compound **10)** is a lactone that was isolated from various *Illicium* species. It was isolated from the leaves of *Illicium parviflorum* Michaux ex Ventenat (Schmidt, 1999) and the pericarps of *Illicium oligandrum* Merr. and Chun (Zhu et al., 2009) and *Illicium dunnianum* Tutcher (Jianmei and Chunshu, 1996).

1,3,6-Tri-O-galloyl-beta-D-glucose (compound **11**) is a glycoside which was isolated from the leaves of *Terminalia bellirica* (Gaertn.) Roxb. (Kim et al., 2018; Manome et al., 2022).

Similarly, 1,2,4,6-tetra-O-galloyl-beta-D-glucose (compound **12**) is another glycoside that was isolated from the leaves of *Phyllanthus emblica* L. (Zhang et al., 2000), the fresh pericarps of *Juglans sigillata* Dode (Si et al., 2011) and the leaves of *Melastoma malabathricum* L. (Yoshida et al., 1982).

Pyrocatechuic acid (compound **13)** is a phenolic acid which was isolated from the ethyl acetate extract of *Mangifera casturi* bark (Pardede and Koketsu, 2016). Pyrocatechuic acid was also isolated from the fruit extract of *Flacourtia inermis* Roxb (George et al., 2011).

Pyrogallol (compound **14)** belongs to the class of phenols and has been previously isolated from the stem and bark of *Barringtonia asiatica* (L.) Kurz (Umaru, 2020). It was also isolated from the rhizomes of *Bergenia ciliata* (Haw.) Sternb (Zafar et al., 2019). In a previous study, the interaction of pyrogallol with AGLUC was evaluated through computational simulations and it was found that pyrogallol inhibits AGLUC with an IC_50_ value of 0.72 ± 0.051 mM (Zheng et al., 2018). Pyrogallol was also found to have an IC_50_ value of 8.7 mM against LOX (Yasumoto, Yamamoto, and Mitsuda, 1970).

There have been no previous studies conducted that quantified antidiabetic properties using the AGLUC inhibition assay, and anti-inflammatory activity using the LOX inhibition assay for compounds **3, 5, 7, and 10–13**.

## Discussion

Asthma and diabetes are two noncommunicable diseases which affect a significant portion of the global population. Glucose metabolism disorders (which ultimately lead to diabetes) have been identified as potential risk factors for the exacerbation of asthma symptoms and the development of severe asthma (Wu, 2020). These disorders cause changes in the lungs similar to those caused by asthma, mainly through a pathway involving insulin excess. Similarly, chronic airway inflammation has been postulated to increase the risk of T2DM. In a large study of middle-aged and older women in the USA, it was found that women with preexisting asthma had a higher risk of developing T2DM, independent of traditional diabetes risk factors (Song et al., 2010).

It has been identified that there is a significant association between the occurrence of asthma and T2DM (Uppal et al., 2023). The NF-κB signaling pathway is believed to cause asthma. NF-κB regulates the expression of certain proinflammatory molecules which cause low-grade inflammation, indicated by increased levels of IL-6, tumor necrosis factor (TNF), C-reactive protein (CRP), and adhesion molecules (Liu, 2007; Edwards et al., 2009, as cited in Uppal et al. (2023)). Low-grade inflammation has been identified as a major contributor to the development of T2DM. It is speculated that the eventual manifestation of diabetes is due to the development of insulin resistance in the liver, smooth muscle, and vascular endothelium influenced by increased circulating levels of certain inflammatory cytokines, which is in turn caused by chronic airway inflammation.

Human arachidonic acid 15-lipoxygenase (ALOX15 or 15-LOX or 12/15-LOX) is a heme-free dioxygenase which catalyzes the formation of hydroperoxy derivatives via the peroxidation of certain polyunsaturated fatty acids. ALOX15 has been found to promote inflammation by metabolizing linoleic acid to 13(S)-hydroperoxyoctadecenoic acid, which then activates NF-κB (Dwarakanath et al., 2004, as cited in He et al. (2023)). It has also been found that ALOX15 metabolites, such as 12S-hydroxyeicosatetraenoic acid, can stimulate the expression of IL-6 and TNF-α in a dose-dependent manner (Wen et al., 2007, as cited in He et al. (2023)), which indicates that ALOX15 can induce inflammatory cascades.

ALOX15 is more highly expressed in airway epithelial cells where it regulates the secretion of mucus, and releases chemokines that act on immune cells, ultimately enhancing pro-inflammatory signaling pathways related to airway inflammatory diseases. In particular, ALOX15 promotes eosinophilic inflammation, the migration of immune cells, and the remodeling of the airway (Xu et al., 2021). It has been hypothesized that ALOX15 inhibitors have the potential to be used as treatments for airway inflammatory diseases, including asthma. ALOX15 and its metabolites have also been implicated in the pathology and mechanism of T2DM, which may be related to the effect of ALOX15 on islet cell and macrophage functions, (He et al., 2023), as well as in serious complications that arise from the disease, such as diabetic retinopathy, peripheral neuropathy, and nephropathy (Singh and Rao, 2019). This implies that ALOX15 inhibitors may also be explored as a possible treatment for T2DM and/or its complications. Compounds or extracts that inhibit both ALOX15 and α-glucosidase might be possible new sources of treatments for T2DM and asthma, especially for women.

In this study, a framework utilizing bioassay-guided fractionation combined with dereplication through UHPLC-MS/MS and database searching was used. Bioactive fractions from *C. intermedia* stem and *D. dao* bark were generated, and dereplication of the bioactive fractions through UHPLC-MS/MS analysis and database searching via GNPS were conducted. A literature search on the putatively identified compounds from dereplication was then performed to see whether there have been previous studies on their antidiabetic and/or anti-inflammatory activities, which might explain the bioactivity of the fractions that were observed in the enzyme inhibition assays.

The putatively identified compounds from CINS are presented in Table 2; Figure 1.

This is the first report of compounds **2** to **7** being putatively identified in *C. intermedia* stem. This is also the first report of compound **1** being putatively identified in *C. intermedia* stem, as it was only previously extracted from the leaves and roots of the plant (Chang et al., 1996).

Compounds **1**, **2**, and **6** were previously reported to exhibit inhibitory activity against AGLUC, with reported IC_50_ values of compounds **1**, **2**, and **6** being 2.18 μg/mL, 2.58 ± 0.08 µM (or 1.63701 ± 0.03 μg/mL), and 30.69 ± 1.47 μg/mL, respectively (You et al., 2012; Pham et al., 2014; Fang et al., 2022). Compounds **4** and **5** have not been previously reported to have inhibitory activity against AGLUC. However, compound **4** has been reported to have inhibitory activity against α-amylase, maltase, and sucrase (Kato et al., 2008). A compound closely related to compound **5**, hyperin, differing by the removal of the galloyl moiety, exhibited inhibitory activity against AGLUC with an IC_50_ of 19.26 μg/mL (Zhang et al., 2013). The previously reported IC_50_ values of compounds **1, 2,** and **6** are all greater compared to the observed IC_50_ value of CINS against AGLUC.

Compounds **2** and **4** were previously reported to exhibit inhibitory activity against LOX, with their reported IC_50_ values being 41 ± 4 µM (or 26.01 ± 2.5 μg/mL) and 15.1 µM (or 2.902 μg/mL), respectively (Deng et al., 2007; Pham et al., 2014).

The putatively identified compounds from the dereplication of DDAB are shown in Table 3; Figure 2, 3.

This is the first report of compounds **6, 8**, and **10–14** being putatively identified in *D. dao* bark.

From the compound hits of the dereplication results of DDAB, only compound **6** was previously reported to exhibit inhibitory activity against AGLUC with an IC_50_ value of 30.69 ± 1.47 μg/mL (Fang et al., 2022). It was previously reported that the methanolic extract from *D. dao* bark has been shown to possess AGLUC inhibitory activity, with an IC_50_ value of 3.24 μg/mL (Yusro et al., 2016).

Only one of the putatively identified compounds from DDAB (compound **14**) was found to have previous studies which quantified their anti-inflammatory activity using the LOX inhibition assay, with an IC_50_ value of 8.7 mM (Yasumoto et al., 1970).

Previous studies have reported IC_50_ values of some of the putatively identified compounds in CINS and DDAB. The differences between IC_50_ values obtained in this study for fractions CINS and DDAB, and IC_50_ values from previous studies on the putatively identified compounds are shown in Table 4.

**TABLE 4:** Differences between IC_50_ values from enzyme inhibition assays on CINS and DDAB and previously reported IC_50_ values of putative hits.

Fraction/Compound	Putatively identified in	IC_50_ (in µg/mL)	
		AGLUC	LOX
CINS	**—**	0.25 ± 0.06	2.57 ± 0.86
DDAB	**—**	0.25 ± 0.04	3.58 ± 0.96
ellagic acid (compound 1)	CINS, DDAB	2.18 You et al. (2012)	N/A
corilagin (compound 2)	CINS	1.63701 ± 0.03 Pham et al. (2014)	26.01 ± 2.5 Pham et al. (2014)
isoscopoletin (compound 4)	CINS	N/A	2.902 Deng et al. (2007)
oleamide (compound 6)	CINS, DDAB	30.69 ± 1.47 Fang et al. (2022)	N/A
pyrogallol (compound 14)	DDAB	N/A	1.097 × 10^3^ Yasumoto et al. (1970)

It is observed that the previously reported IC_50_ values of the putative compounds are greater than the obtained IC_50_ values for CINS and DDAB. This implies that the compounds in fractions CINS and DDAB might be acting in synergy to inhibit AGLUC and LOX at lower concentrations. In addition, other compounds present in CINS and DDAB that were unidentified through dereplication may also be responsible for the observed activity of the fractions against AGLUC and LOX. It can be observed in the chromatogram traces of CINS and DDAB (Supplementary Figures S2–S3, S5–S6) that not all peaks are accounted for when compared to the putative hits from the dereplication results. Further fractionation of these active fractions may lead to more potent bioactivities observed in the enzyme assays since the fractions still contain multiple compounds. Further purification of the bioactive fractions is also necessary to ascertain if the bioactivity observed for the fractions is due to a synergistic effect or if an unidentified compound is responsible for the observed bioactivity.

From the dereplication compound hits and the literature search that was conducted, corilagin (compound **2**) was identified as the only compound having been previously reported to be active against both AGLUC and LOX, having IC_50_ values of 2.58 ± 0.08 µM against AGLUC and 41 ± 4 µM against LOX (Pham et al., 2014). Corilagin (compound **2**) is a gallotannin that has been extensively studied and was shown to exhibit several pharmacological activities (Li et al., 2018).

Corilagin was found to significantly reduce the production of some proinflammatory cytokines and mediators and was also found to reduce cyclooxygenase-2 expression at both the protein and gene level (Zhao et al., 2008, as cited in Li et al. (2018)). Streptozotocin-induced diabetic rats that were treated orally with corilagin were shown to have reduced fasting blood glucose levels compared to the diabetic control rats at the end of the study, similar to the level by which glibenclamide reduced the FBG levels (Nandini and Naik, 2019). Corilagin was found to reduce airway inflammation and collagen deposition in ovalbumin-induced asthmatic mice via the adenosine monophosphate-activated protein kinase pathway (Jin and Yi, 2023). In mice fed with a high fat diet (HFD) to induce nonalcoholic fatty liver disease (NAFLD), treatment with corilagin was found to reduce HFD-induced accumulation of fat in the liver and liver injury, improved plasma lipid concentrations, as well as improve other metabolic disorders associated with NAFLD, such as glucose intolerance and insulin resistance in HFD-fed mice (Liao et al., 2022). Corilagin has been found to exhibit antitumor activities against different cancer cell lines, both *in vitro* and *in vivo* (Li et al., 2018). Corilagin was also found to inhibit the binding of the spike receptor binding domain (spike-RBD) of SARS-CoV-2 virus to human angiotensin-converting enzyme 2 (hACE2) in a dose-dependent manner (Yang et al., 2021). This indicates that corilagin interferes with the fusion of spike-RBD and hACE2 and implies that corilagin may be considered as a potential candidate as an inhibitor for the entry of SARS-CoV-2. In safety tests, corilagin was found to have had almost no toxic effects on normal cells or tissues (Li et al., 2018).

Based on these previous findings, it is strongly recommended that corilagin be further studied as a potential new treatment for people living with diabetes and/or asthma, especially for women, and perhaps for other diseases as well, such as NAFLD, cancer, and COVID-19.

It must be acknowledged that the current study has several limiting factors. First, the samples which were tested (CINS and DDAB) for their bioactivity against AGLUC and LOX are only subfractions from plant extracts, and not purified compounds. The identity of the compound/s from the subfractions which are responsible for the observed bioactivity against the bioassays conducted cannot be confirmed with absolute certainty at this point in time. It is recommended that the active compound/s responsible for the bioactivity are purified and isolated further in future studies.

Second, the study only used *in vitro* enzymatic assays to determine the bioactivity of the samples, in contrast to *in vivo*, *ex vivo*, or *in vitro* cell based assays. In our research group, only *in vitro* enzymatic assays are carried out for all samples. *In vitro* assays, in general, are faster and require less of the sample to work with, but *in vivo* assays are preferred because they more closely mimic clinical conditions while also providing toxicity data (Sarker and Nahar, 2012). It is recommended that the bioactive subfractions that were identified in this study, as well as the putatively identified compounds from these subfractions, should be tested against *in vivo* bioassays (or *in vitro* cell-based assays) for their anti-diabetic, anti-inflammatory, or anti-asthmatic activity. Multiple animal models can be used for *in vivo* testing of the anti-diabetic (King, 2012), anti-inflammatory (Patil et al., 2019), and anti-asthmatic (Kianmeher et al., 2016) activity of a compound or an extract.

Finally, although compounds from CINS and DDAB were identified, the identities of these compounds are only putative at this point. Dereplication by LC-MS/MS does not provide information on the configuration or constitution of a molecule based solely on the molecular ion match or fragmentation pattern match (Zani and Carroll, 2017). Hyphenated techniques also do not consider configurational isomerism within a molecule. The information gathered from dereplication using LC-MS/MS data by database searching is also limited by the compounds and spectra which are actually listed in the database. For example, the compound libraries hosted on GNPS which are used for spectral matching contain a multitude of compounds and reference spectra (221,000 reference library spectra from 18,163 compounds) (Wang, et al., 2016). However, it can still be limiting in the sense that it cannot account for the majority of compounds which may be produced by terrestrial plants. A single plant species is estimated to produce between 5,000 to tens of thousands of compounds, and collectively all plant species are estimated to produce between 100,000 and 1,000,000 compounds (Fang et al., 2019). Again, further purification of the identified bioactive subfractions must be carried out, along with structural identification using other characterization techniques to establish the identity of the bioactive component/s.

## Conclusion

This study aimed to determine potential anti-diabetic and anti-inflammatory bioactive hits from *C. intermedia* stem and *D. dao* bark. Bioactive fractions from *C. intermedia* stem and *D. dao* bark were generated, and dereplication through UHPLC-MS/MS and database searching was performed. Seven compounds were putatively identified from the *C. intermedia* stem active fraction, and six of these compounds were putatively identified from this plant for the first time. Nine compounds were putatively identified from the *D. dao* bark active fraction, and seven of these compounds were putatively identified from this plant for the first time. One putative compound from the *C. intermedia* stem active fraction (corilagin) has been previously reported to have inhibitory activity against both AGLUC and LOX. It is suggested that corilagin should be prioritized in further studies on new therapeutics which target the treatment of T2DM and/or asthma because of its dual inhibitory activity against AGLUC and LOX, as well as its several beneficial pharmacological activities and low reported toxicity.

## Data Availability

The data presented in the study are deposited in Figshare. This data can be found here: https://doi.org/10.6084/m9.figshare.25272313.v1.

## References

[B1] Abaya L. M. M. Villaseñor I. M. (2019). “Gravity column chromatography (GCC),” in *Tuklas Lunas Protocols for drug Discovery and development manual 1: Collection and Extraction of Biosources and Purification of bioactives*. . Editors Alvero R. G. Y. Guevara A. P. (Taguig: Philippine Council for Health Research and Development), 52–55.

[B2] Adair L. S. Kuzawa C. McDade T. Carba D. B. Borja J. B. (2018). Seventeen-year changes in body mass index, waist circumference, elevated blood pressure, and diabetes phenotypes in a cohort of Filipino women. Asia Pac. J. Public Health 30, 561–571. 10.1177/1010539518800366 30221978 PMC6263034

[B3] Alara O. R. Abdurahman N. H. Ali H. A. (2023). Optimization of microwave-enhanced extraction parameters to recover phenolic compounds and antioxidants from Corchorus olitorius leaves. Chem. Zvesti 77 (8), 4217–4233. 10.1007/s11696-023-02771-x PMC10088688 37362792

[B4] Allanigue E. Hernandez C. (2019). Tuklas Lunas protocols for drug discovery and development: manual 2B primary bioactivity assays. Taguig City, Philippines: Philippine Council for Health Research and Development, 151–165.

[B5] Allard P.-M. Péresse T. Bisson J. I. Gindro K. Marcourt L. Pham V. C. (2016). Integration of molecular networking and in-silico MS/MS fragmentation for natural products dereplication. Anal. Chem. 88 (6), 3317–3323. 10.1021/acs.analchem.5b04804 26882108

[B116] Atanasov A. G. Waltenberger B. Pferschy-Wenzig E. -M. Linder T. Wawrosch C. Uhrin P. (2015). Discovery and resupply of pharmacologically active plant-derived natural products: a review. Biotechnol. Adv. 33, 1582–1614. 10.1016/j.biotechadv.2015.08.001 26281720 PMC4748402

[B6] Auerbach B. J. Kiely J. S. Cornicelli J. A. (1992). A spectrophotometric microtiter-based assay for the detection of hydroperoxy derivatives of linoleic acid. Anal. Biochem. 201 (2), 375–380. 10.1016/0003-2697(92)90354-a 1632527

[B7] Axelrod B. Cheesbrough T. M. Laakso S. (1981). [53] Lipoxygenase from soybeans: EC 1.13.11.12 Linoleate:oxygen oxidoreductase. Methods Enzym., 441–451. 10.1016/0076-6879(81)71055-3

[B8] BenSaad L. A. Kim K. H. Quah C. C. Kim W. R. Shahimi M. (2017). Anti-inflammatory potential of ellagic acid, gallic acid and punicalagin A&B isolated from Punica granatum. BMC Complement. Altern. Med. 17, 47. 10.1186/s12906-017-1555-0 28088220 PMC5237561

[B10] Céspedes C. L. Avila J. G. Martínez A. Serrato B. Calderón-Mugica J. C. Salgado-Garciglia R. (2006). Antifungal and antibacterial activities of Mexican tarragon (Tagetes lucida). J. Agric. Food Chem. 54, 3521–3527. 10.1021/jf053071w 19127719

[B11] Chang Y. Lin M.-S. Jiang R.-L. Huang S.-C. Ho L. (1996). 20-Epibryonolic acid, phytosterols and ellagic acid from Coriaria intermedia. Phytochemistry 42 (2), 559–560. 10.1016/0031-9422(95)00935-3

[B12] Chen J.-J. Chen P.-H. Liao C.-H. Huang S.-Y. Chen I.-S. (2007). New phenylpropenoids, bis(1-phenylethyl)phenols, bisquinolinone alkaloid, and anti-inflammatory constituents from Zanthoxylum integrifoliolum. J. Nat. Prod. 70, 1444–1448. 10.1021/np070186g 17822293

[B13] Chen X. Zhu Q. Jia Z. (2001). Pregnane glycoside, lignan glycosides, triterpene glycosyl ester and flavonoid glycosides from Rubus amabilis. Planta Med. 67, 270–273. 10.1055/s-2001-11996 11345701

[B14] Chowdhury N. U. Guntur V. P. Newcomb D. C. Wechsler M. E. (2021). Sex and gender in asthma. Eur. Respir. Rev. 30 (162), 210067. 10.1183/16000617.0067-2021 34789462 PMC8783601

[B117] Clemen-Pascual L. M. Macahig R. A. S. Rojas N. R. L. (2022). Comparative toxicity, phytochemistry, and use of 53 Philippine medicinal plants. Toxicol. Rep. 9, 22–35. 10.1016/j.toxrep.2021.12.002 34976744 PMC8685920

[B15] Colombo R. De L. Batista A. N. Teles H. L. Silva G. H. Bomfim G. C. C. (2009). Validated HPLC method for the standardization of Phyllanthus niruri (herb and commercial extracts) using corilagin as a phytochemical marker. Biomed. Chromatogr. 23, 573–580. 10.1002/bmc.1155 19277954

[B16] Dapar M. L. G. (2021). “Dracontomelon dao (blanco) Merr. and Rolfe Anacardiaceae,” in Ethnobotany of the mountain regions of southeast Asia ethnobotany of mountain regions. Editor Franco F. M. (Cham: Springer International Publishing), 373–378. 10.1007/978-3-030-38389-3_79

[B17] Deng S. Palu A. K. West B. J. Su C. X. Zhou B.-N. Jensen J. C. (2007). Lipoxygenase inhibitory constituents of the fruits of noni (*Morinda citrifolia*) collected in tahiti. J. Nat. Prod. 70, 859–862. 10.1021/np0605539 17378609

[B18] Diabetes (2023). Available at: https://www.who.int/news-room/fact-sheets/detail/diabetes (Accessed November 14, 2023).

[B19] Dwarakanath R. S. Sahar S. Reddy M. A. Castanotto D. Rossi J. J. Natarajan R. (2004). Regulation of monocyte chemoattractant protein-1 by the oxidized lipid, 13-hydroperoxyoctadecadienoic acid, in vascular smooth muscle cells via nuclear factor-kappa B (NF-kappa B). J. Mol. Cell. Cardiol. 36 (4), 585–595. 10.1016/j.yjmcc.2004.02.007 15081318

[B20] Edwards M. R. Bartlett N. W. Clarke D. Birrell M. Belvisi M. Johnston S. L. (2009). Targeting the NF-kappaB pathway in asthma and chronic obstructive pulmonary disease. Pharmacol. Ther. 121 (1), 1–13. 10.1016/j.pharmthera.2008.09.003 18950657 PMC7172981

[B21] Fang C. Fernie A. R. Luo J. (2019). Exploring the diversity of plant metabolism. Trends Plant Sci. 24 (1), 83–98. 10.1016/j.tplants.2018.09.006 30297176

[B22] Fang H.-L. Liu M.-L. Li S.-Y. Song W.-Q. Ouyang H. Xiao Z.-P. (2022). Identification, potency evaluation, and mechanism clarification of α-glucosidase inhibitors from tender leaves of Lithocarpus polystachyus Rehd. Food Chem. 371, 131128. 10.1016/j.foodchem.2021.131128 34563970

[B23] Feng-Lin H. Jhy-Yih C. (1993). Phenolics from Tectaria subtriphylla. Phytochemistry 34, 1625–1627. 10.1016/S0031-9422(00)90858-6

[B24] García-Menaya J. M. Cordobés-Durán C. García-Martín E. Agúndez J. A. G. (2019). Pharmacogenetic factors affecting asthma treatment response. Potential implications for drug therapy. Front. Pharmacol. 10, 520. 10.3389/fphar.2019.00520 31178722 PMC6537658

[B25] George S. Benny P. Kuriakose S. George C. (2011). Antibiotic activity of 2, 3-dihydroxybenzoic acid isolated from Flacourtia inermis fruit against multidrug resistant bacteria. Asian J. Pharm. Clin. Res. 4, 126–130.

[B26] Good R. D. (1930). The geography of the genus coriaria. New Phytol. 29, 170–198. 10.1111/j.1469-8137.1930.tb06989.x

[B27] Guri A. J. Evans N. P. Hontecillas R. Bassaganya-Riera J. (2011). T cell PPARγ is required for the anti-inflammatory efficacy of abscisic acid against experimental IBD. J. Nutr. Biochem. 22, 812–819. 10.1016/j.jnutbio.2010.06.011 21109419 PMC3117068

[B28] Guron M. A. Napaldet J. T. (2020). Distribution and morpho-anatomical characterization of ‘beket’ (Coriaria japonica subsp. intermedia (Matsum) T. C. Huanh) in Cordillera central range, northern Philippines. J. Mt. Sci. 17, 2136–2147. 10.1007/s11629-020-6110-7

[B29] He K. Zhou X. Du H. Zhao J. Deng R. Wang J. (2023). A review on the relationship between Arachidonic acid 15-Lipoxygenase (ALOX15) and diabetes mellitus. PeerJ 11, e16239. 10.7717/peerj.16239 37849828 PMC10578307

[B31] Hubert J. Nuzillard J.-M. Renault J.-H. (2015). Dereplication strategies in natural product research: how many tools and methodologies behind the same concept? Phytochem. Rev. 16 (1), 55–95. 10.1007/s11101-015-9448-7

[B32] Jeon S. H. Kim N. Ju Y.-J. Gee M. S. Lee D. Lee J. K. (2020). Phytohormone abscisic acid improves memory impairment and reduces neuroinflammation in 5xFAD mice by upregulation of LanC-like protein 2. IJMS 21, 8425. 10.3390/ijms21228425 33182586 PMC7697599

[B33] Jianmei H. Chunshu Y. (1996). Pseudoanisatin-like sesquiterpene lactones from the pericarps of Illicium dunnianum. Phytochemistry 42, 1375–1376. 10.1016/0031-9422(95)00903-5

[B34] Jin Y. Yi C. (2023). Corilagin attenuates airway inflammation and collagen deposition in ovalbumin-induced asthmatic mice. Allergologia Immunopathol. 51 (6), 97–103. 10.15586/aei.v51i6.988 37937502

[B120] Jung H. A. Park J. J. Islam Md. N. Jin S. E. Min B. -S. Lee J. -H. (2012). Inhibitory activity of coumarins from Artemisia capillaris against advanced glycation end product formation. Arch. Pharm. 35, 1021–1035. 10.1007/s12272-012-0610-0 22870812

[B35] Junio H. A. Garcia K. G. Rellin K. F. B. (2019). “Ultra high-performance liquid chromatography with high-resolution mass spectrometry (UHPLC-HRMS)-Based metabolomics for dereplication,” in *Tuklas Lunas® Protocols for drug Discovery and development manual 4: structure identification, synthesis, and Derivatization of bioactives* . Editors Alvero R. G. Y. Guevara A. P. (Taguig: Philippine Council for Health Research and Development), 2–30.

[B36] Kato A. Minoshima Y. Yamamoto J. Adachi I. Watson A. A. Nash R. J. (2008). Protective effects of dietary chamomile tea on diabetic complications. J. Agric. Food Chem. 56, 8206–8211. 10.1021/jf8014365 18681440

[B37] Kaul K. Tarr J. M. Kohner’ E. M. Chibber’ R. (2023). Introduction to diabetes mellitus. 10.1007/978-1-4614-5441-0_1 23393665

[B38] Khan M. R. Omoloso A. D. (2002). Antibacterial and antifungal activities of Dracontomelon dao. Fitoterapia 73 (4), 327–330. 10.1016/s0367-326x(02)00076-x 12234577

[B39] Kianmeher M. Ghorani V. Boskabady M. H. (2016). Animal model of asthma, various methods and measured parameters: a methodological review. Iran. J. Allergy Asthma Immunol. 15 (6), 445–465. 28129678

[B40] Kim M.-S. Lee D. Y. Lee J. Kim H. W. Sung S. H. Han J.-S. (2018). Terminalia chebula extract prevents scopolamine-induced amnesia via cholinergic modulation and anti-oxidative effects in mice. BMC Complement. Altern. Med. 18, 136. 10.1186/s12906-018-2212-y 29716575 PMC5930767

[B41] Kimura Y. Suzuki A. Takematsu T. Konnai M. Takeuchi Y. (1982). (+)-Abscisic acid and two compounds showing chlorophyll degradation activity in Cuscuta pentagona engelm. Agric. Biol. Chem. 46, 1071–1073. 10.1271/bbb1961.46.1071

[B42] King A. J. (2012). The use of animal models in diabetes research. Br. J. Pharmacol. 166 (3), 877–894. 10.1111/j.1476-5381.2012.01911.x 22352879 PMC3417415

[B43] Klein B. E. Klein R. Moss S. E. (1999). Mortality and hormone-related exposures in women with diabetes. Diabetes Care 22, 248–252. 10.2337/diacare.22.2.248 10333941

[B44] Kumar V. Prakash O. Kumar S. Narwal S. (2011). α-glucosidase inhibitors from plants: a natural approach to treat diabetes. Pharmacogn. Rev. 5 (9), 19–29. 10.4103/0973-7847.79096 22096315 PMC3210010

[B45] Kuo S.-Y. Hsieh T.-J. Wang Y.-D. Lo W.-L. Hsui Y.-R. Chen C.-Y. (2008). Cytotoxic constituents from the leaves of Cinnamomum subavenium. Chem. Pharm. Bull. 56, 97–101. 10.1248/cpb.56.97 18175985

[B46] Li X. Deng Y. Zheng Z. Huang W. Chen L. Tong Q. (2018). Corilagin, a promising medicinal herbal agent. Biomed. Pharmacother. 99, 43–50. 10.1016/j.biopha.2018.01.030 29324311

[B47] Li Y. Xia H. Wu M. Wang J. Lu X. Wei S. (2017). Evaluation of the antibacterial effects of flavonoid combination from the leaves of Dracontomelon dao by microcalorimetry and the quadratic rotary combination design. Front. Pharmacol. 8, 70. 10.3389/fphar.2017.00070 28261101 PMC5313536

[B48] Liao M. Zhang R. Wang Y. Mao Z. Wu J. Guo H. (2022). Corilagin prevents non-alcoholic fatty liver disease via improving lipid metabolism and glucose homeostasis in high fat diet-fed mice. Front. Nutr. 9, 983450. 10.3389/fnut.2022.983450 36071929 PMC9443665

[B49] Liu H. Zhang J. Liu L. Lian G. Shi R. Xu M. (2023). Global disease burden and attributable risk factor analysis of asthma in 204 countries and territories from 1990 to 2019. Allergy, Asthma Immunol. Res. 15 (4), 473–495. 10.4168/aair.2023.15.4.473 37153981 PMC10359648

[B50] Liu S. Tinker L. Song Y. Rifai N. Bonds D. E. Cook N. R. (2007). A prospective study of inflammatory cytokines and diabetes mellitus in a multiethnic cohort of postmenopausal women. Archives Intern. Med. 167 (15), 1676–1685. 10.1001/archinte.167.15.1676 17698692

[B51] Manome T. Hara Y. Ahmed F. Sadhu S. K. Ishibashi M. (2022). Thannilignan glucoside and 2-(β-glucopyranosyl)-3-isoxazolin-5-one derivative, two new compounds isolated from Terminalia bellirica. J. Nat. Med. 76, 482–489. 10.1007/s11418-021-01593-z 35040087

[B52] Molino R. J. E. J. Rellin K. F. B. Nellas R. B. Junio H. A. (2021). Small in size, big on taste: metabolomics analysis of flavor compounds from Philippine garlic. PLOS ONE 16 (5), e0247289. 10.1371/journal.pone.0247289 34014935 PMC8136657

[B53] Moon S.-M. Lee S.Ah. Hong J. H. Kim J.-S. Kim D. K. Kim C. S. (2018). Oleamide suppresses inflammatory responses in LPS-induced RAW264.7 murine macrophages and alleviates paw edema in a carrageenan-induced inflammatory rat model. Int. Immunopharmacol. 56, 179–185. 10.1016/j.intimp.2018.01.032 29414648

[B54] Naeem A. Silveyra P. (2019). Sex differences in paediatric and adult asthma. Eur. Med. J. 4, 27–35. 10.33590/emj/10312930 PMC6641536 31328173

[B55] Naing M. Yerro J. Fernandez P. Amor E. (2019). Tuklas Lunas protocols for drug discovery and development: manual 2B primary bioactivity assays. Taguig City, Philippines: Philippine Council for Health Research and Development, 2–16.

[B56] Nandini H. S. Naik P. R. (2019). Action of corilagin on hyperglycemia, hyperlipidemia and oxidative stress in streptozotocin-induced diabetic rats. Chemico-Biological Interact. 299, 186–193. 10.1016/j.cbi.2018.12.012 30582900

[B118] Nie Y. Yang W. Liu Y. Yang J. Lei X. Gerwick W. H. (2020). Acetylcholinesterase inhibitors and antioxidants mining from marine fungi: bioassays, bioactivity coupled LC–MS/MS analyses and molecular networking. MLST 2 (4), 386–397. 10.1007/s42995-020-00065-9

[B57] Nishimura T. Wang L.-Y. Kusano K. Kitanaka S. (2005). Flavonoids that mimic human ligands from the whole plants of Euphorbia lunulata. Chem. Pharm. Bull. 53, 305–308. 10.1248/cpb.53.305 15744103

[B58] Nothias L.-F. Petras D. Schmid R. Dührkop K. Rainer J. Sarvepalli A. (2020). Feature-based molecular networking in the GNPS analysis environment. Nat. Methods 17 (9), 905–908. 10.1038/s41592-020-0933-6 32839597 PMC7885687

[B59] Nyamboki D. K. Bedane K. G. Hassan K. Brieger L. Strohmann C. Spiteller M. (2021). Cytotoxic compounds from the stem bark of two subsp. of Bersama abyssinica. J. Nat. Prod. 84 (5), 1453–1458. 10.1021/acs.jnatprod.0c01141 33974421

[B60] Oh Y. T. Lee J. Y. Lee J. Lee J. H. Kim J.-E. Ha J. (2010). Oleamide suppresses lipopolysaccharide-induced expression of iNOS and COX-2 through inhibition of NF-kappaB activation in BV2 murine microglial cells. Neurosci. Lett. 474, 148–153. 10.1016/j.neulet.2010.03.026 20298753

[B61] Okuda T. Yoshida T. Mori K. (1975). Brevifolin, corilagin and other phenols from Geranium thunbergii. Phytochemistry 14, 1877–1878. 10.1016/0031-9422(75)85321-0

[B62] Padhi S. Nayak A. K. Behera A. (2020). Type II diabetes mellitus: a review on recent drug based therapeutics. Biomed. Pharmacother. 131, 110708. 10.1016/j.biopha.2020.110708 32927252

[B63] Panche A. N. Diwan A. D. Chandra S. R. (2016). Flavonoids: an overview. J. Nutr. Sci. 5 (47), e47. 10.1017/jns.2016.41 28620474 PMC5465813

[B64] Pardede A. Koketsu M. (2016). Antioxidant and antileukemic activity of chemical components from bark of Mangifera casturi. Comp. Clin. Pathol. 26 (3), 499–504. 10.1007/s00580-016-2387-x

[B65] Park J. Y. Kim J. Y. Son Y. G. Kang S. D. Lee S. W. Kim K. D. (2023). Characterization of chemical composition and antioxidant activity of Eucalyptus globulus leaves under different extraction conditions. Appl. Sci. 13, 9984. 10.3390/app13179984

[B66] Patil K. R. Mahajan U. B. Unger B. S. Goyal S. N. Belemkar S. Surana S. J. (2019). Animal models of inflammation for screening of anti-inflammatory drugs: implications for the discovery and development of phytopharmaceuticals. Int. J. Mol. Sci. 20 (18), 4367. 10.3390/ijms20184367 31491986 PMC6770891

[B67] Peña J. F. D. Dapar M. L. G. Aranas A. T. Cabrido C. K. Torres M. A. J. Demayo C. G. (2019). ASSESSMENT of antimicrobial, antioxidant and cytotoxic properties of the ethanolic extract from Dracontomelon dao (blanco) Merr. Rolfe 10, 18–29.

[B68] Pennington E. Yaqoob Z. J. Al-kindi S. G. Zein J. (2019). Trends in asthma mortality in the United States: 1999 to 2015. Am. J. Respir. Crit. Care Med. 199 (12), 1575–1577. 10.1164/rccm.201810-1844le 30917289 PMC6835077

[B69] Pham A. T. Malterud K. E. Paulsen B. S. Diallo D. Wangensteen H. (2014). α-Glucosidase inhibition, 15-lipoxygenase inhibition, and brine shrimp toxicity of extracts and isolated compounds from Terminalia macroptera leaves. Pharm. Biol. 52, 1166–1169. 10.3109/13880209.2014.880486 24635511

[B70] Philippine Council for Health Research and Development, Department of Science and Technology (2024). Tuklas lunas the philippine drug discovery and development program*.* [brochure]. Available at: https://www.healthresearch.ph/index.php/about-pnhrs/downloads/category/222-technologies?download=1025:tuklas-lunas-program.

[B71] Punzalan C. V. Villaseñor I. M. (2019a). “Extraction and solvent partitioning,” in *Tuklas Lunas® Protocols for drug Discovery and development manual 1: Collection and Extraction of Biosources and Purification of bioactives* . Editors Alvero R. G. Y. Guevara A. P. (Taguig: Philippine Council for Health Research and Development), 34–36.

[B72] Punzalan C. V. Villaseñor I. M. (2019b). “Vacuum liquid chromatography (VLC),” in *Tuklas Lunas® Protocols for drug Discovery and development manual 1: Collection and Extraction of Biosources and Purification of bioactives* . Editors Alvero R. G. Y. Guevara A. P. (Taguig: Philippine Council for Health Research and Development), 44–48.

[B73] Ragasa C. Y. Batarra T. C. Vivar J. L. A. Reyes M. M. D. L. Shen C.-C. (2017). Chemical Constituents of Dracontomelon Dao (Blanco) Merr. et Rolfe. Rolfe. PJ 9, 654–656. 10.5530/pj.2017.5.103

[B74] Ragasa C. Y. Vivar J. L. A. van Altena I. A. (2016). Secondary metabolites from Dracontomelon dao (Merr. and Rolfe). 8.

[B75] Reed J. Bain S. Kanamarlapudi V. (2021). A review of current trends with type 2 diabetes epidemiology, aetiology, pathogenesis, treatments and future perspectives. Diabetes, Metabolic Syndrome Obes. Targets Ther. 14 (1), 3567–3602. 10.2147/dmso.s319895 PMC8369920 34413662

[B76] Ríos J.-L. Giner R. Marín M. Recio M. (2018). A pharmacological update of ellagic acid. Planta Med. 84, 1068–1093. 10.1055/a-0633-9492 29847844

[B77] Rodrigues M. J. Custódio L. Lopes A. Oliveira M. Neng N. R. Nogueira J. M. F. (2017). Unlocking the *in vitro* anti-inflammatory and antidiabetic potential of Polygonum maritimum. Pharm. Biol. 55, 1348–1357. 10.1080/13880209.2017.1301493 28301958 PMC6130642

[B78] Sadeghian H. Jabbari A. (2015). 15-Lipoxygenase inhibitors: a patent review. Expert Opin. Ther. Pat. 26 (1), 65–88. 10.1517/13543776.2016.1113259 26560362

[B119] Salac E. L. O. Alvarez M. R. Gaurana R. S. Grijaldo S. J. Serrano L. M. De Juan F. (1969). Local plants as potential sources of tannins in Egypt, part. IV (Aceraceae to Flacourtiaceae). Plant Food Hum. Nutr. 11, 1–15. 10.3390/plants11182380

[B79] Saleh N. A. M. Sherbeiny A. E. A. Sissi H. I. (1969). Local plants as potential sources of tannins in Egypt, part. IV (Aceraceae to Flacourtiaceae). Plant Food Hum. Nutr. 17, 384–394. 10.1007/BF01100201

[B80] San Luis G. D. Balangcod T. D. Abucay J. B. Wong F. M. Balangcod K. D. Afifi I. G. (2014). Phytochemical and antimicrobial screening of indigenous species that have potential for revegetation of landslides in Atok, Benguet, Philippines. Indian J. Traditional Knowl. 13 (1), 56–62.

[B81] Sarker S. D. Nahar L. (2012). “An introduction to natural products isolation,” in Natural products isolation. Editors Sarker S. D. Nahar L. (New York, NY 10013, USA: Humana Press), 1–25. 10.1007/978-1-61779-624-1_1 22367891

[B82] Schmidt T. J. (1999). Novel s eco -prezizaane sesquiterpenes from North American Illicium species. J. Nat. Prod. 62, 684–687. 10.1021/np980382a 10346945

[B84] Shobana S. Sreerama Y. N. Malleshi N. G. (2009). Composition and enzyme inhibitory properties of finger millet (Eleusine coracana L.) seed coat phenolics: mode of inhibition of α-glucosidase and pancreatic amylase. Food Chem. 115 (4), 1268–1273. 10.1016/j.foodchem.2009.01.042

[B121] Si C. -L. Zhang Y. Zhu Z. -Y. Liu S. -C. (2011). Chemical constituents with antioxidant activity from the pericarps of Juglans sigillata. Chem. Nat. Compd. 47 (3), 442–445. 10.1007/s10600-011-9956-7

[B85] Singh N. K. Rao G. N. (2019). Emerging role of 12/15-Lipoxygenase (ALOX15) in human pathologies. Prog. Lipid Res. 73, 28–45. 10.1016/j.plipres.2018.11.001 30472260 PMC6338518

[B86] Song Y. Klevak A. Manson J. E. Buring J. E. Liu S. (2010). Asthma, chronic obstructive pulmonary disease, and type 2 diabetes in the Women’s Health Study. Diabetes Res. Clin. Pract. 90 (3), 365–371. 10.1016/j.diabres.2010.09.010 20926152 PMC2993844

[B87] Sun H. Saeedi P. Karuranga S. Pinkepank M. Ogurtsova K. Duncan B. B. (2021). IDF diabetes Atlas: global, regional and country-level diabetes prevalence estimates for 2021 and projections for 2045. Diabetes Res. Clin. Pract. 183, 109119. 10.1016/j.diabres.2021.109119 34879977 PMC11057359

[B88] Sun N.-J. Ho D. K. Hu X. E. Sneddon J. M. Stephens R. E. Cassady J. M. (1995). New cytotoxic fatty acid from Desmos cochinchinensis (annonaceae). Nat. Product. Lett. 7, 35–41. 10.1080/10575639508043184

[B90] Tsugawa H. Cajka T. Kind T. Ma Y. Higgins B. Ikeda K. (2015). MS-DIAL: data-independent MS/MS deconvolution for comprehensive metabolome analysis. Nat. Methods 12 (6), 523–526. 10.1038/nmeth.3393 25938372 PMC4449330

[B91] Umaru I. J. (2020). Pyrogallol isolation, characterization, cytotoxicity, antioxidant and bioactive potentials on selected bacterial and fungi. Int. J. Pharm. Biomed. Res. 7 (2), 1–11. 10.18782/2394-3726.1088

[B92] Uppal P. Mohammed S. A. Rajashekar S. Ravindran S. G. Kakarla M. Gambo M. A. (2023). Type 2 diabetes mellitus and asthma: pathomechanisms of their association and clinical implications. Cureus 15 (3), e36047. 10.7759/cureus.36047 37056543 PMC10089620

[B93] Wang M. Carver J. J. Phelan V. V. Sanchez L. M. Garg N. Peng Y. (2016). Sharing and community curation of mass spectrometry data with global natural products social molecular networking. Nat. Biotechnol. 34, 828–837. 10.1038/nbt.3597 27504778 PMC5321674

[B94] Wen J. Xu Z. Ma X. Zhao Y. (2022). Wound healing effects of Dracontomelon dao on bacterial infection wounds in rats and its potential mechanisms under simulated space environment. Evidence-Based Complementary Altern. Med. 2022, 4593201–4593215. 10.1155/2022/4593201 PMC9249481 35783508

[B95] Wen Y. Gu J. Chakrabarti S. K. Aylor K. Marshall J. Takahashi Y. (2007). The role of 12/15-lipoxygenase in the expression of interleukin-6 and tumor necrosis factor-alpha in macrophages. Endocrinology 148 (3), 1313–1322. 10.1210/en.2006-0665 17170102

[B96] World Health Organization (2023). *Asthma*. WHO. Available at: https://www.who.int/news-room/fact-sheets/detail/asthma (Accessed November 23, 2023).

[B97] Wu T. D. (2020). Diabetes, insulin resistance, and asthma: a review of potential links. Curr. Opin. Pulm. Med. 27 (1), 29–36. 10.1097/mcp.0000000000000738 33002990

[B98] Wu Y. Qu W. Geng D. Liang J.-Y. Luo Y.-L. (2012). Phenols and flavonoids from the aerial part of Euphorbia hirta. Chin. J. Nat. Med. 10, 40–42. 10.1016/S1875-5364(12)60009-0 23302529

[B99] Xu L. Li Y. Dai Y. Peng J. (2018). Natural products for the treatment of type 2 diabetes mellitus: Pharmacology and mechanisms. Pharmacol. Res. 130, 451–465. 10.1016/j.phrs.2018.01.015 29395440

[B100] Xu X. Li J. Zhang Y. Zhang L. (2021). Arachidonic acid 15-lipoxygenase: effects of its expression, metabolites, and genetic and epigenetic variations on airway inflammation. Allergy, Asthma and Immunol. Res. 13 (5), 684–696. 10.4168/aair.2021.13.5.684 34486255 PMC8419644

[B101] Yang J. Y. Sanchez L. M. Rath C. M. Liu X. Boudreau P. D. Bruns N. (2013). Molecular networking as a dereplication strategy. J. Nat. Prod. 76, 1686–1699. 10.1021/np400413s 24025162 PMC3936340

[B102] Yang L. J. Chen R. H. Hamdoun S. Coghi P. Ng J. P. L. Zhang D. W. (2021). Corilagin prevents SARS-CoV-2 infection by targeting RBD-ACE2 binding. Phytomedicine 87, 153591. 10.1016/j.phymed.2021.153591 34029937 PMC8098048

[B103] Yasumoto K. Yamamoto A. Mitsuda H. (1970). Effect of phenolic antioxidants on lipoxygenase reaction. Agric. Biol. Chem. 34 (8), 1162–1168. 10.1080/00021369.1970.10859748

[B104] Yazaki K. Shida S. Okuda T. (1989). Galloylhomoarbutin and related polyphenols from Pyrola incarnata. Phytochemistry 28, 607–609. 10.1016/0031-9422(89)80060-3

[B105] Yoshida T. Seno K. Takama Y. Okuda T. (1982). Bergenin derivatives from Mallotus japonicus. Phytochemistry 21, 1180–1182. 10.1016/S0031-9422(00)82451-6

[B106] You Q. Chen F. Wang X. Jiang Y. Lin S. (2012). Anti-diabetic activities of phenolic compounds in muscadine against alpha-glucosidase and pancreatic lipase. LWT - Food Sci. Technol. 46, 164–168. 10.1016/j.lwt.2011.10.011

[B107] Yusro F. Ohtani K. Kubota S. (2016). Inhibition of α-glucosidase by methanol extracts from wood bark of Anacardiaceae, fabaceae, malvaceae and phyllanthaceae plants family in west kalimantan, Indonesia. 9, 108–122.

[B108] Zafar R. Ullah H. Zahoor M. Sadiq A. (2019). Isolation of bioactive compounds from Bergenia ciliata (haw.) Sternb rhizome and their antioxidant and anticholinesterase activities. BMC Complementary Altern. Med. 19 (1), 296. 10.1186/s12906-019-2679-1 PMC6833214 31694704

[B109] Zani C. L. Carroll A. R. (2017). Database for rapid dereplication of known natural products using data from MS and fast NMR experiments. J. Nat. Prod. 80 (6), 1758–1766. 10.1021/acs.jnatprod.6b01093 28616931

[B110] Zhang X. Liu Z. Bi X. Liu J. Li W. Zhao Y. (2013). Flavonoids and its derivatives from Callistephus chinensis flowers and their inhibitory activities against alpha-glucosidase. EXCLI J. 12, 956–966. 10.17877/DE290R-11527 27298611 PMC4904746

[B111] Zhang Y.-J. Abe T. Tanaka T. Yang C.-R. Kouno I. (2002). Two new acylated flavanone glycosides from the leaves and branches of Phyllanthus emblica. Chem. Pharm. Bull. 50 (6), 841–843. 10.1248/cpb.50.841 12045344

[B112] Zhang Y.-J. Tanaka T. Iwamoto Y. Yang C.-R. Kouno I. (2000). Novel norsesquiterpenoids from the roots of Phyllanthus emblica. J. Nat. Prod. 63, 1507–1510. 10.1021/np000135i 11087593

[B113] Zhao L. Zhang S.-L. Tao J.-Y. Pang R. Jin F. Guo Y.-J. (2008). Preliminary exploration on anti-inflammatory mechanism of Corilagin (beta-1-O-galloyl-3,6-(R)-hexahydroxydiphenoyl-d-glucose) *in vitro* . Int. Immunopharmacol. 8 (7), 1059–1064. 10.1016/j.intimp.2008.03.003 18486919

[B114] Zheng L. Lee J. Yue L.-M. Lim G. T. Yang J.-M. Ye Z.-M. (2018). Inhibitory effect of pyrogallol on α-glucosidase: integrating docking simulations with inhibition kinetics. Int. J. Biol. Macromol. 112, 686–693. 10.1016/j.ijbiomac.2018.02.026 29425876

[B115] Zhu Q. Tang C.-P. Ke C.-Q. Wang W. Zhang H.-Y. Ye Y. (2009). Sesquiterpenoids and phenylpropanoids from pericarps of Illicium oligandrum. J. Nat. Prod. 72, 238–242. 10.1021/np8004979 19159273

