# Supplementary material for: Anti-diabetic and anti-inflammatory bioactive hits from Coriaria intermedia Matsum. stem and Dracontomelon dao (Blanco) Merr. & Rolfe bark through bioassay-guided fractionation and liquid chromatography-tandem mass spectrometry
Source: Front Pharmacol. 2024 Mar 8;15:1349725. doi: 10.3389/fphar.2024.1349725 (PMC10957545; doi:10.3389/fphar.2024.1349725)
Supplement: Supplementary file 1 [file DataSheet1.pdf]

Supplementary Material

1 Supplementary Figures and Tables

1.1 Supplementary Figures

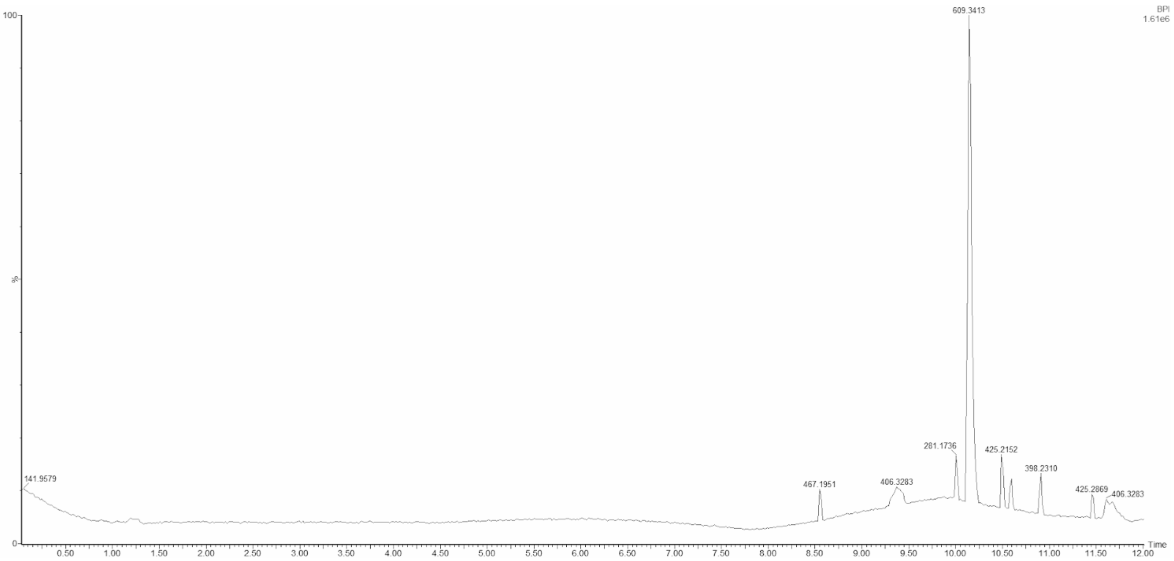

Supplementary Figure 1. Base peak intensity (BPI) chromatogram of blank ran in UHPLC-QToF (positive mode).

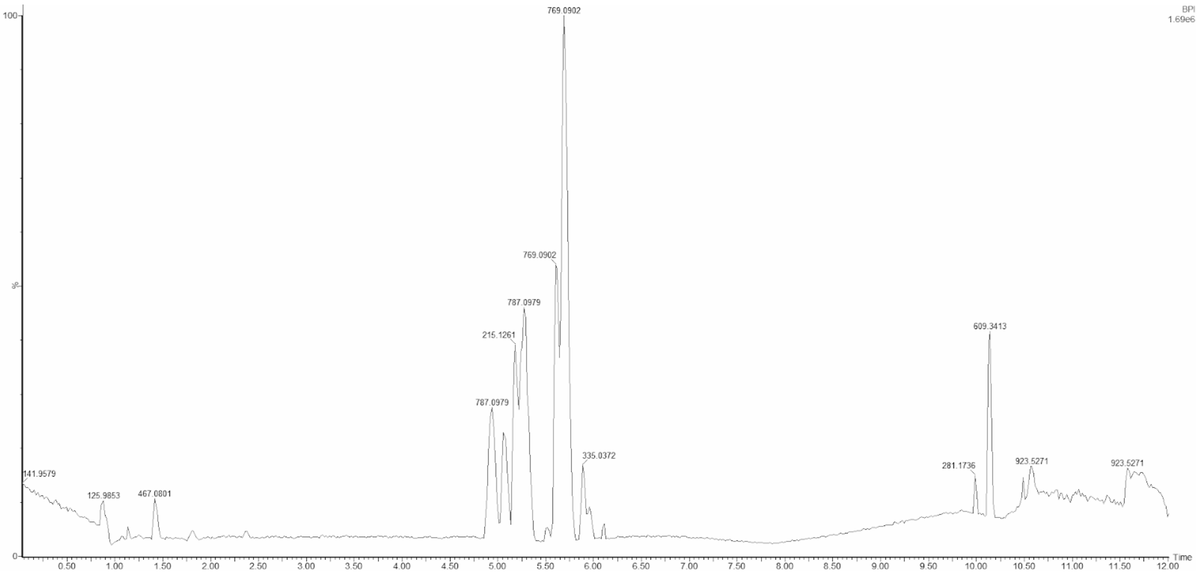

Supplementary Figure 2. Base peak intensity (BPI) chromatogram of CINS ran in UHPLC-QToF (positive mode).

Supplementary Material

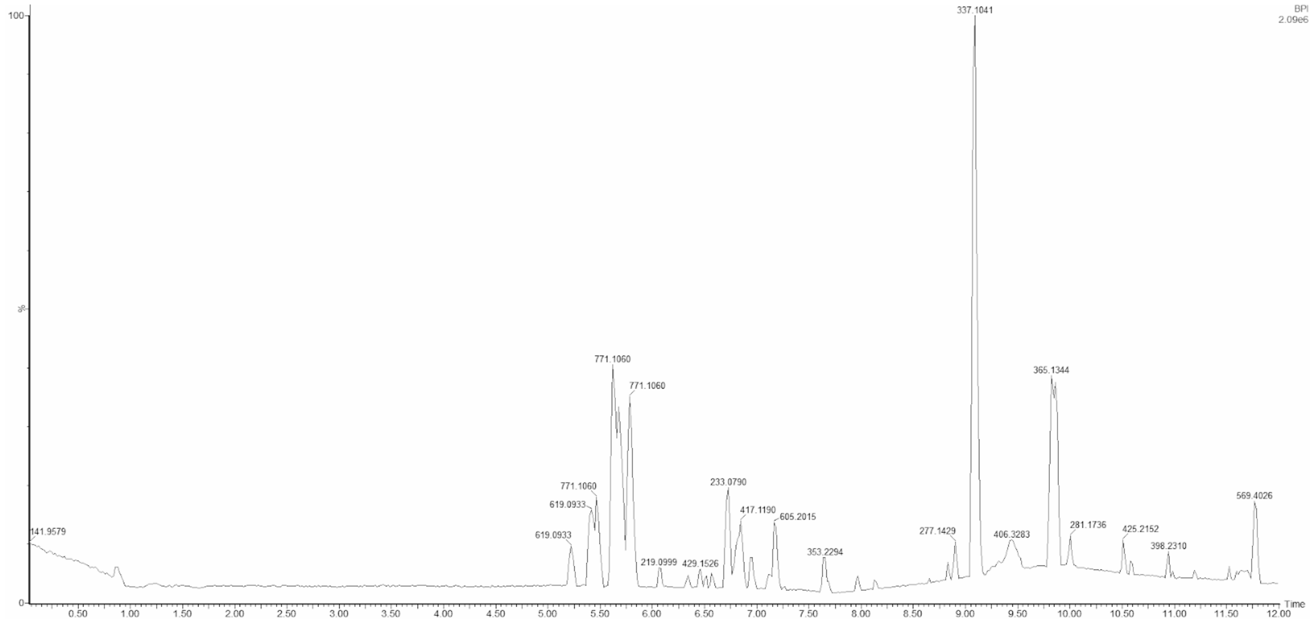

Supplementary Figure 3. Base peak intensity (BPI) chromatogram of DDAB ran in UHPLC-QToF (positive mode).

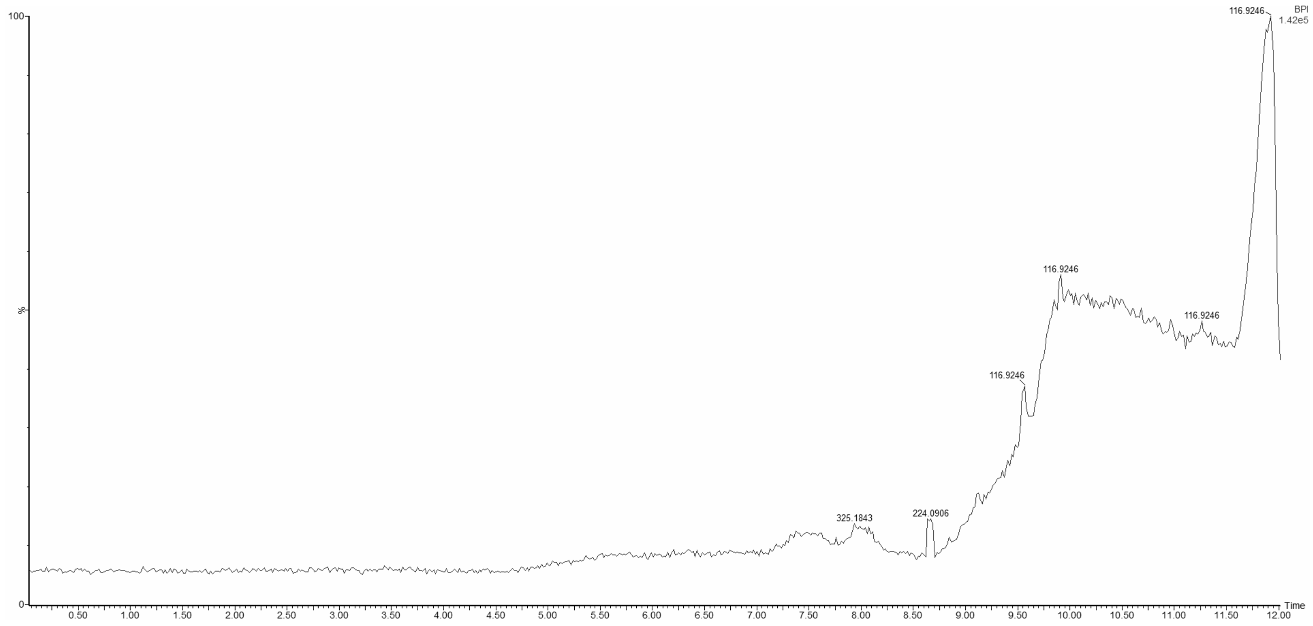

Supplementary Figure 4. Base peak intensity (BPI) chromatogram of blank ran in UHPLC-QToF (negative mode).

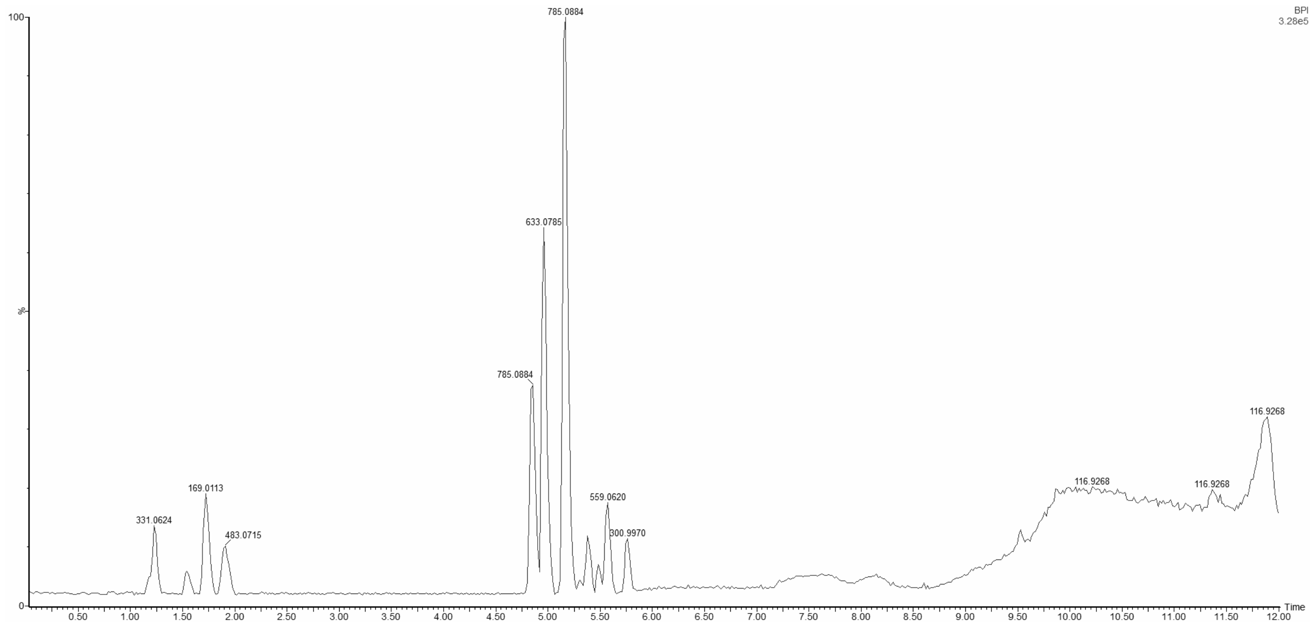

Supplementary Figure 5. Base peak intensity (BPI) chromatogram of CINS ran in UHPLC-QToF (negative mode).

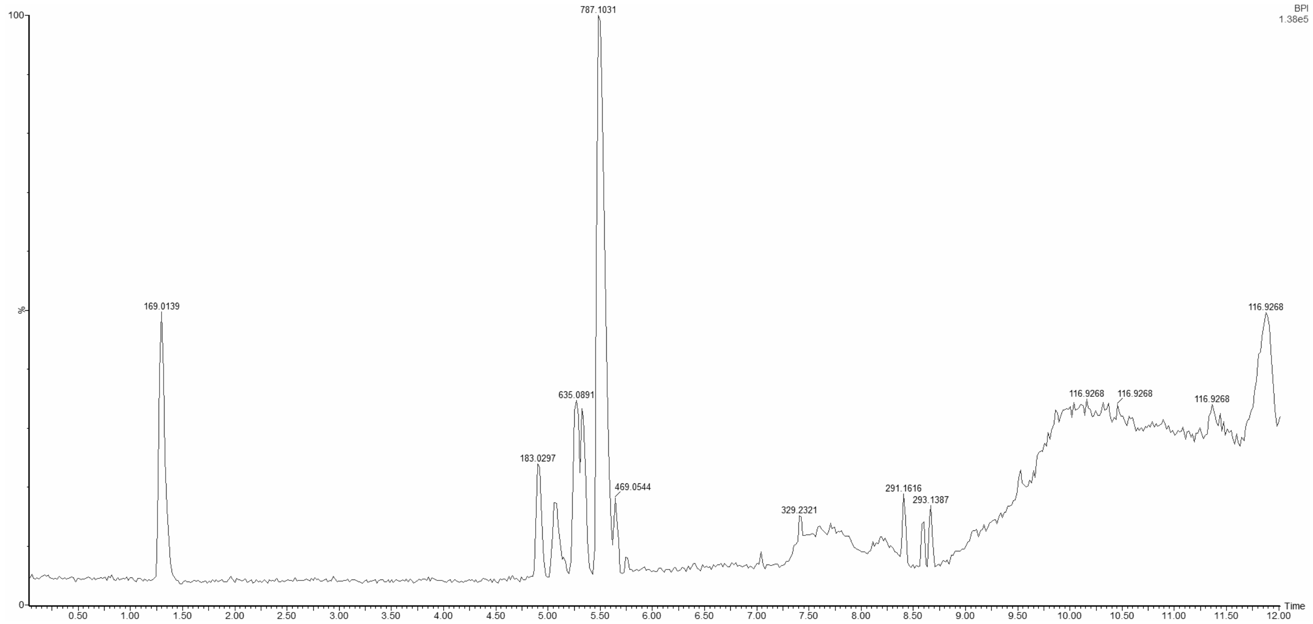

Supplementary Figure 6. Base peak intensity (BPI) chromatogram of DDAB ran in UHPLC-QToF (negative mode).

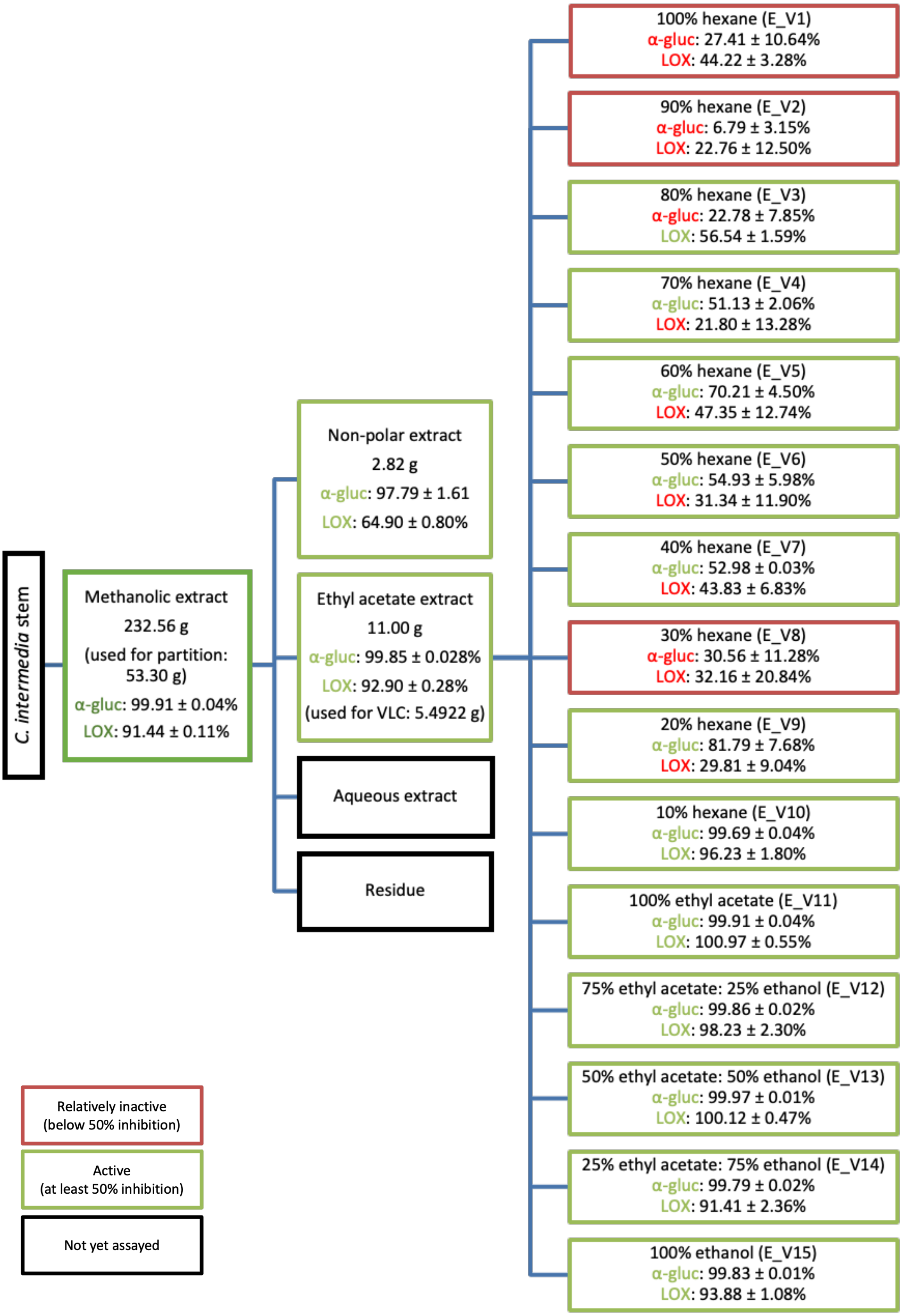

Supplementary Figure 7. Schematic diagram of the vacuum liquid (VLC) chromatography fractionation of *C. intermedia* stem ethyl acetate extract.

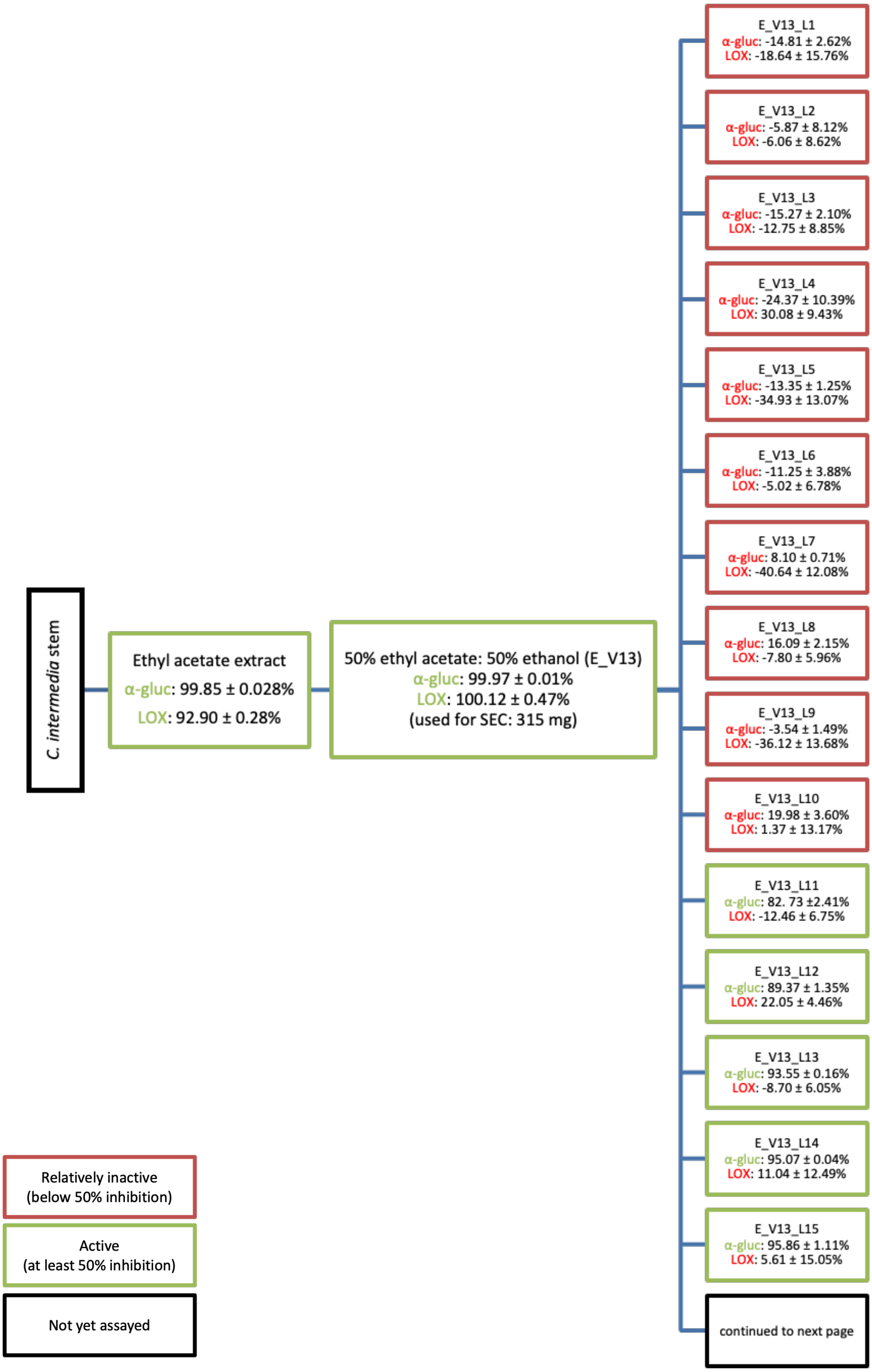

Supplementary Figure 8. Size-exclusion chromatography (SEC) fractionation of *C. intermedia* stem E_V13.

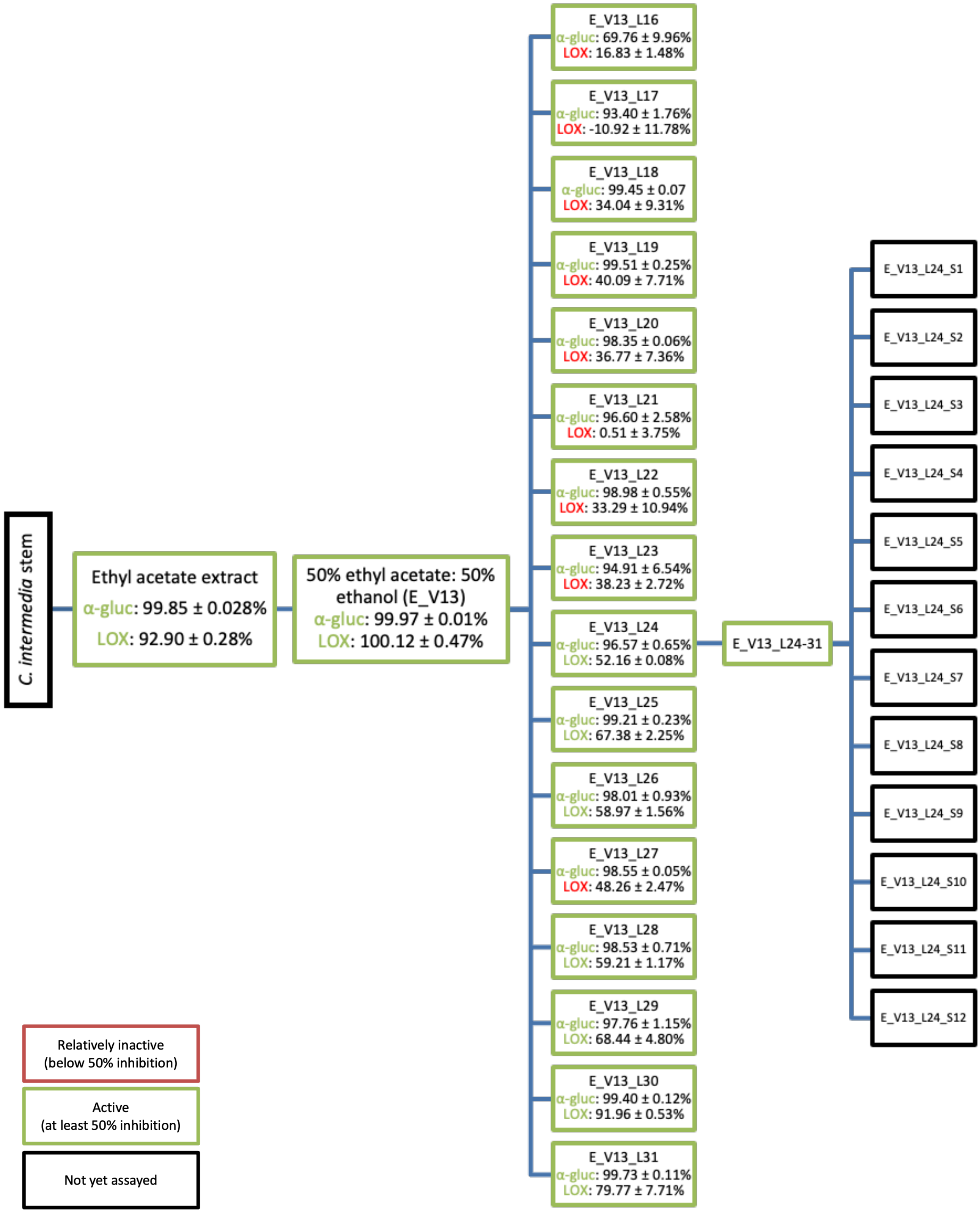

Supplementary Figure 9. Size-exclusion chromatography (SEC) fractionation of *C. intermedia* stem E_V13 and solid-phase extraction fractionation (SPE) of E_V13_L24-31 (pooled).

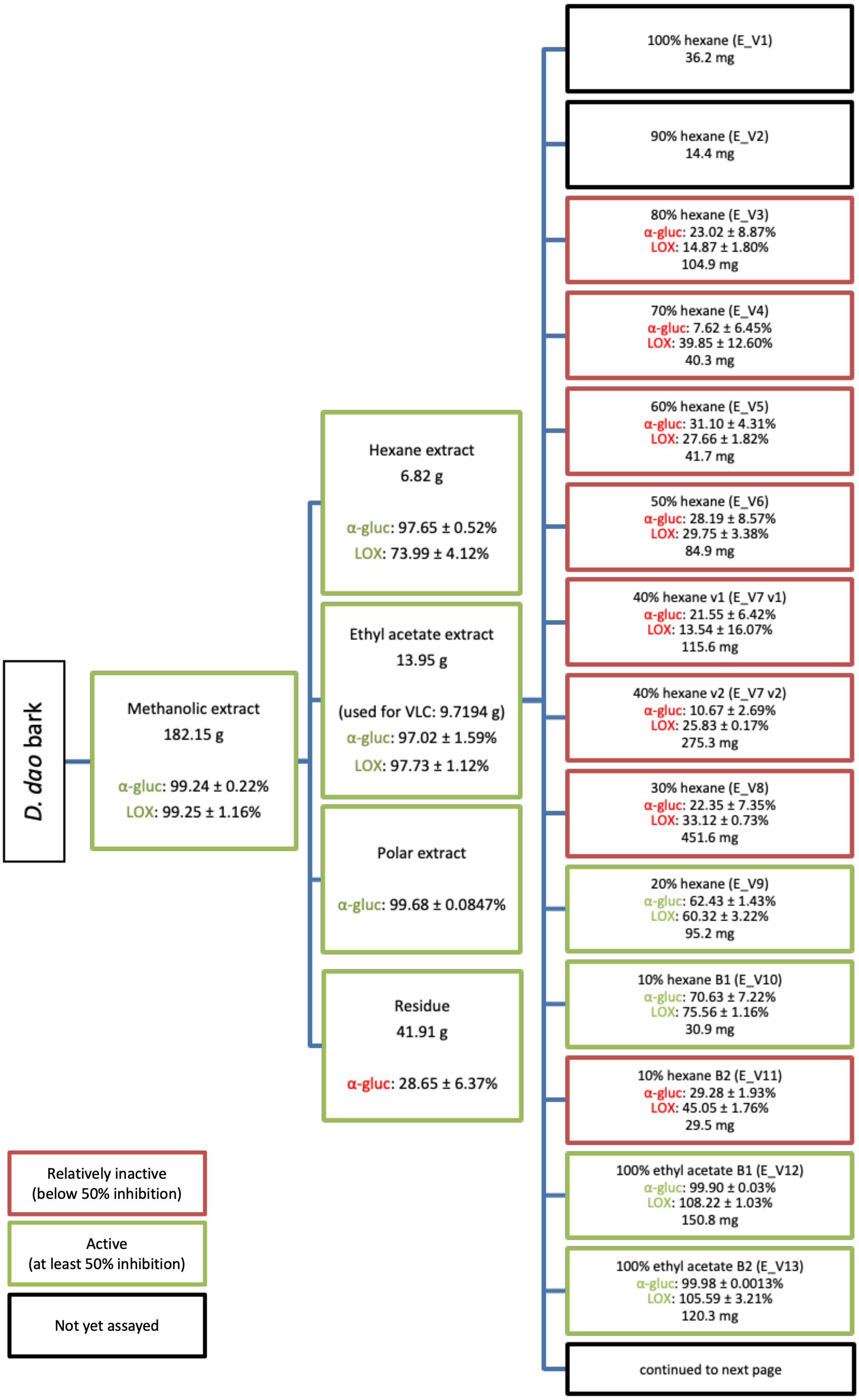

Supplementary Figure 10. Schematic diagram of the vacuum liquid (VLC) chromatography fractionation of *D. dao* bark ethyl acetate extract.

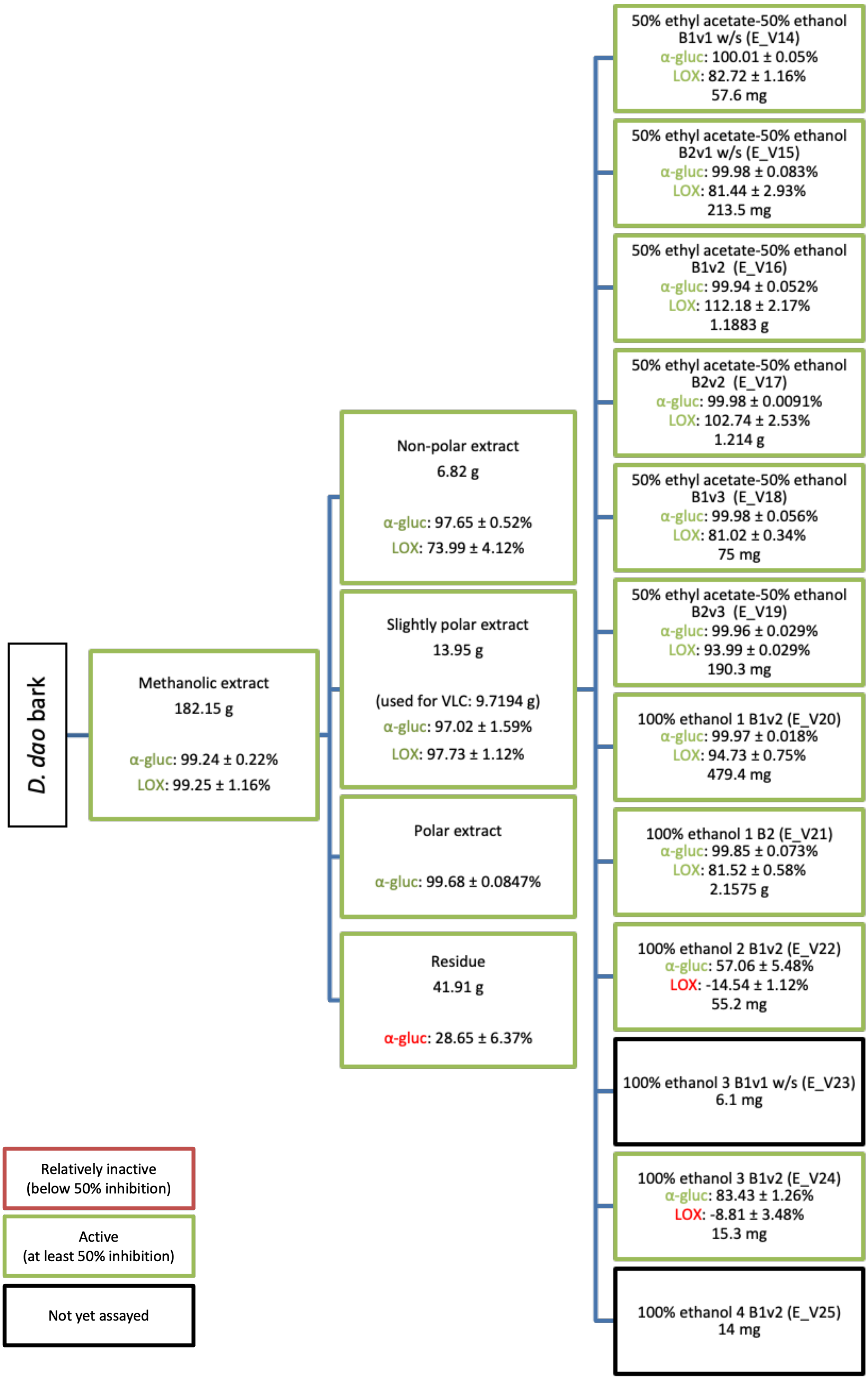

Supplementary Figure 11. Schematic diagram of the vacuum liquid (VLC) chromatography fractionation of *D. dao* bark ethyl acetate extract.

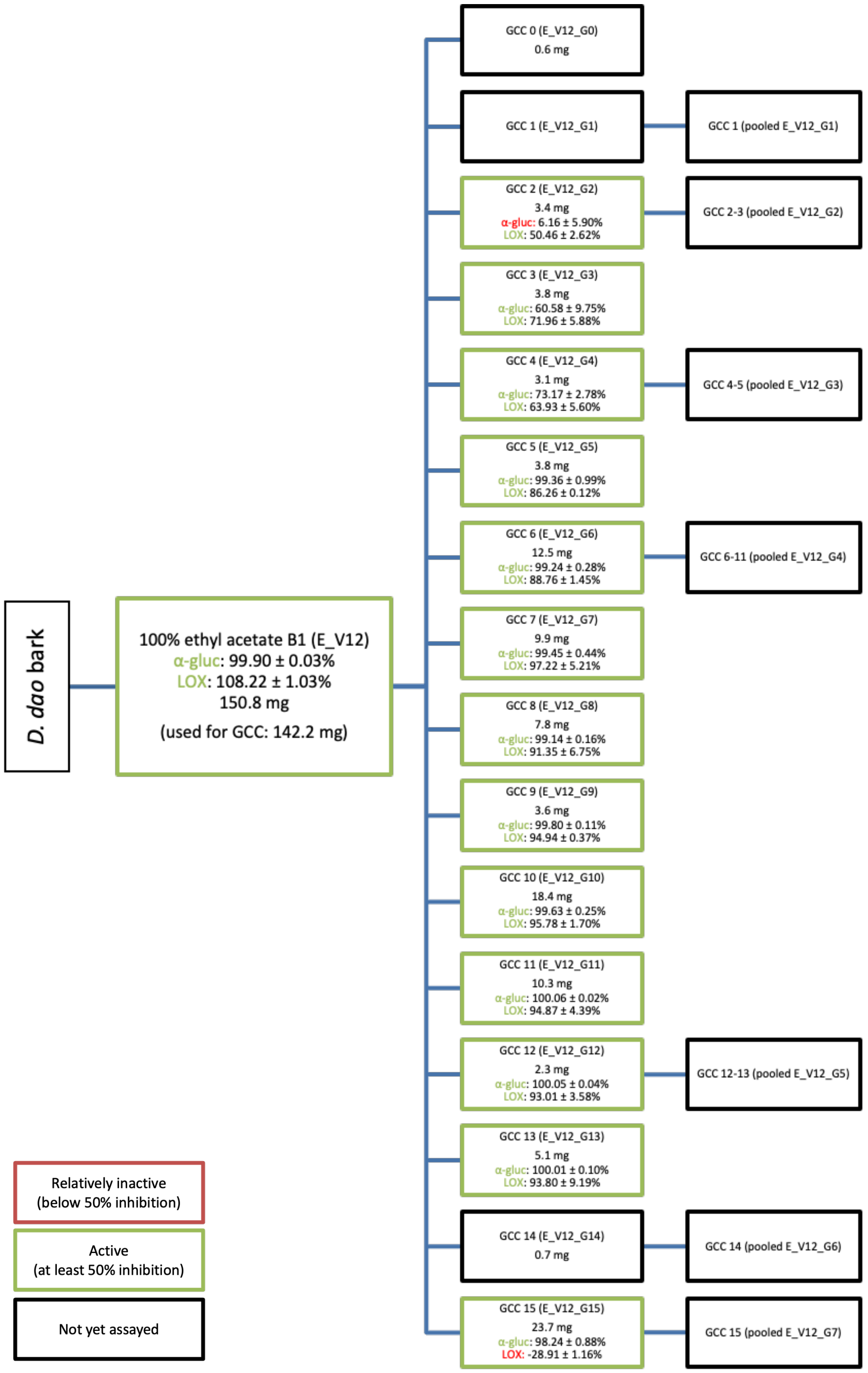

Supplementary Figure 12. Gravity column chromatography (GCC) fractionation of *D. dao* bark E_V12.

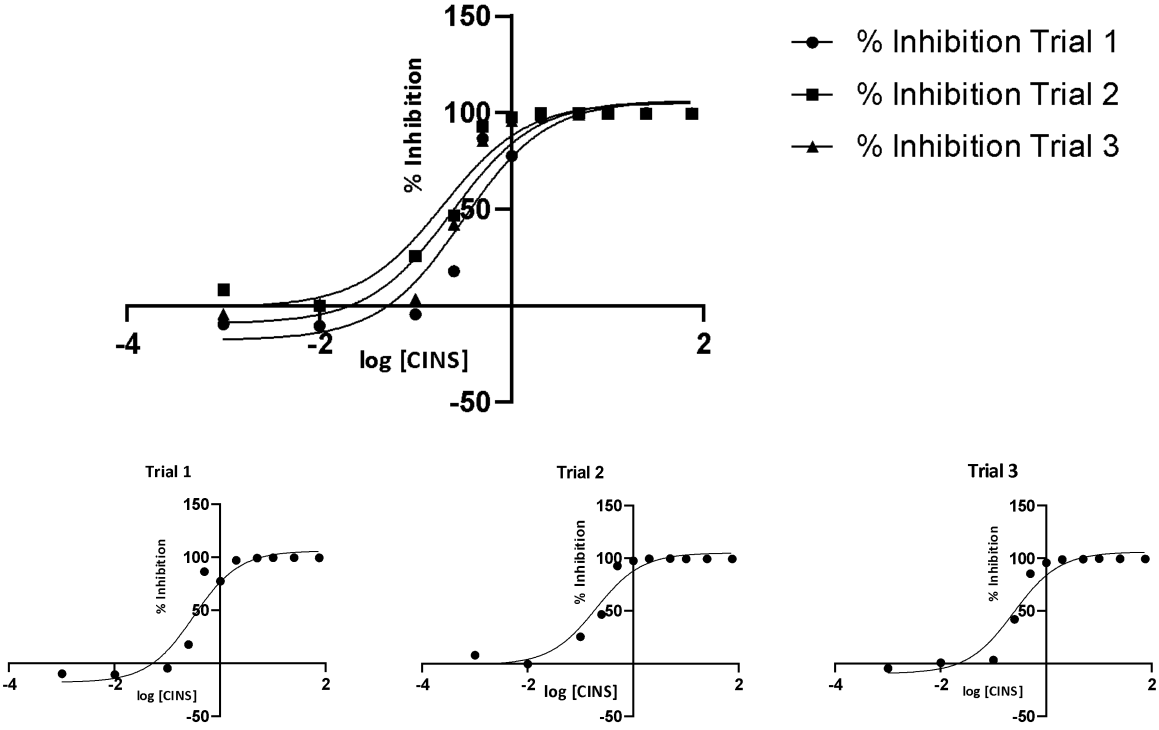

Supplementary Figure 13. Dose-response curves of CINS against AGLUC.

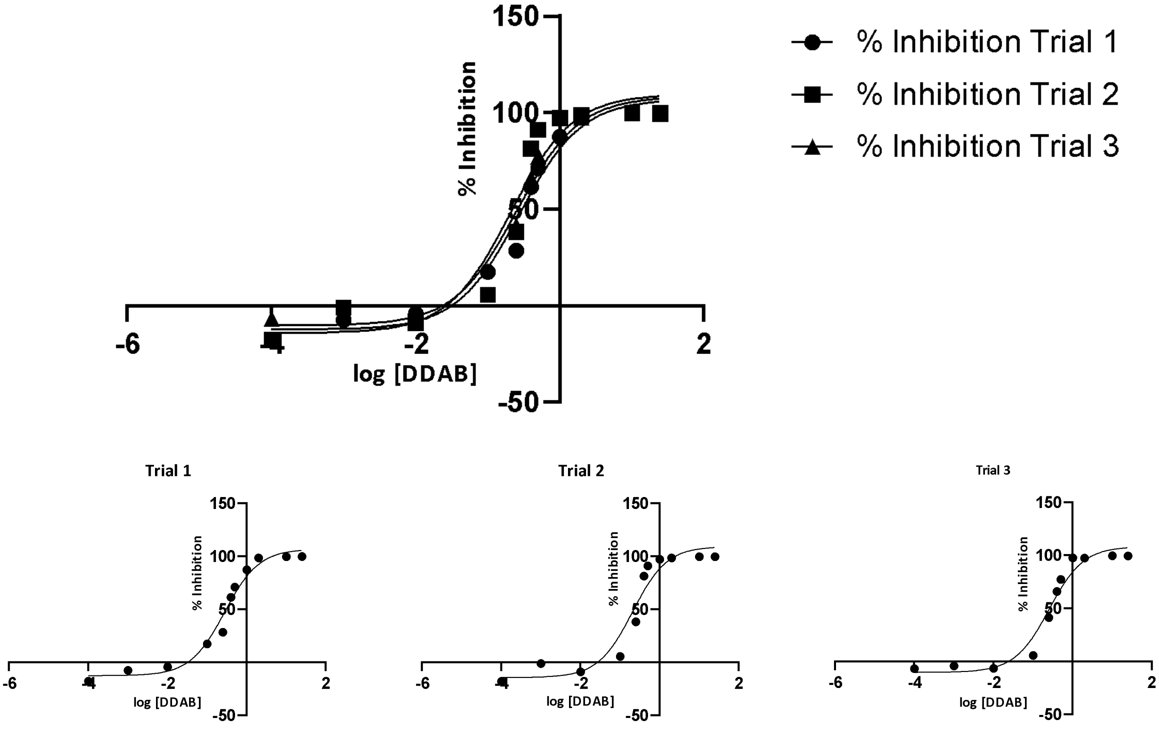

Supplementary Figure 14. Dose-response curves of DDAB against AGLUC.

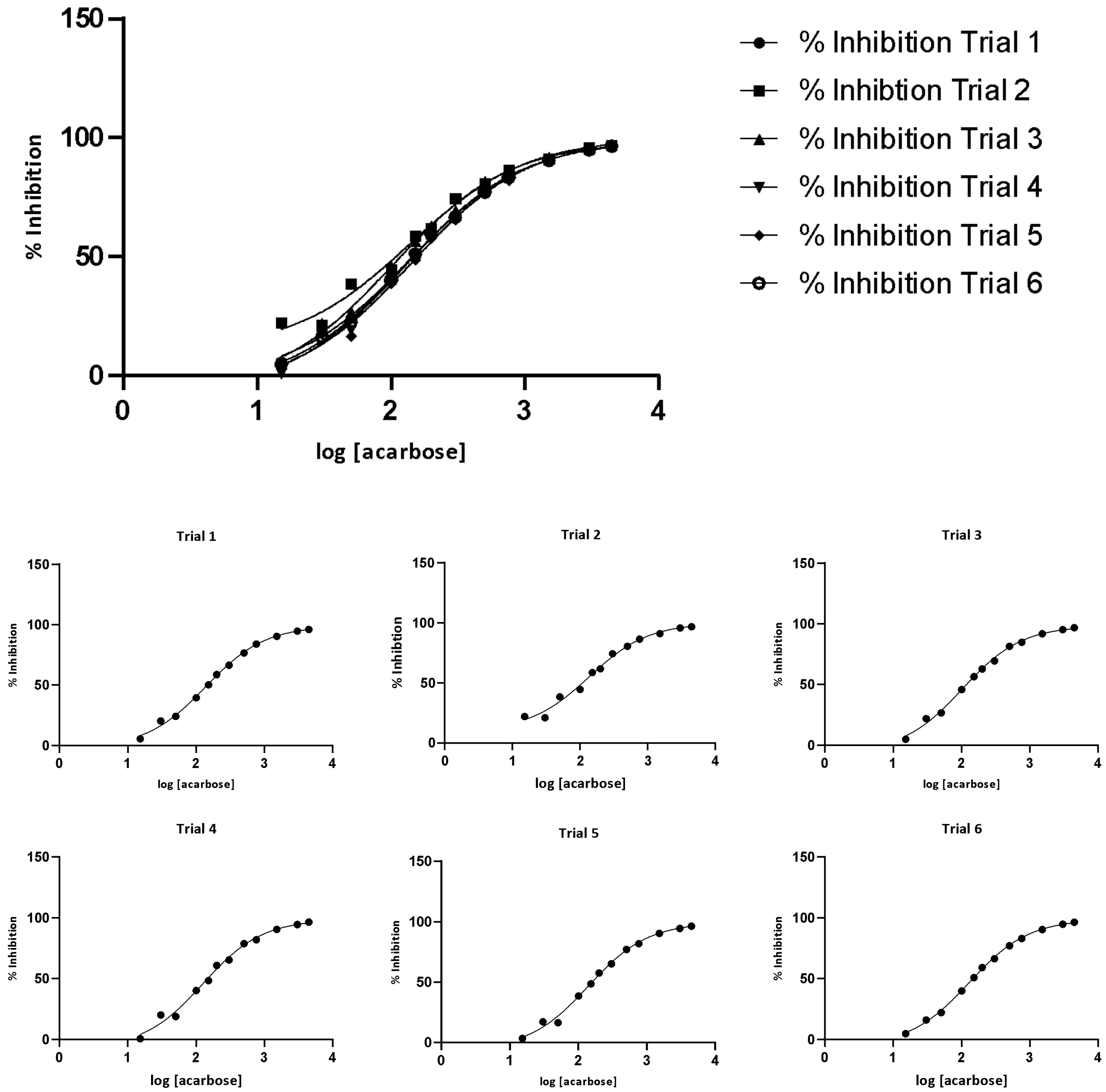

Supplementary Figure 15. Dose-response curves of acarbose against AGLUC.

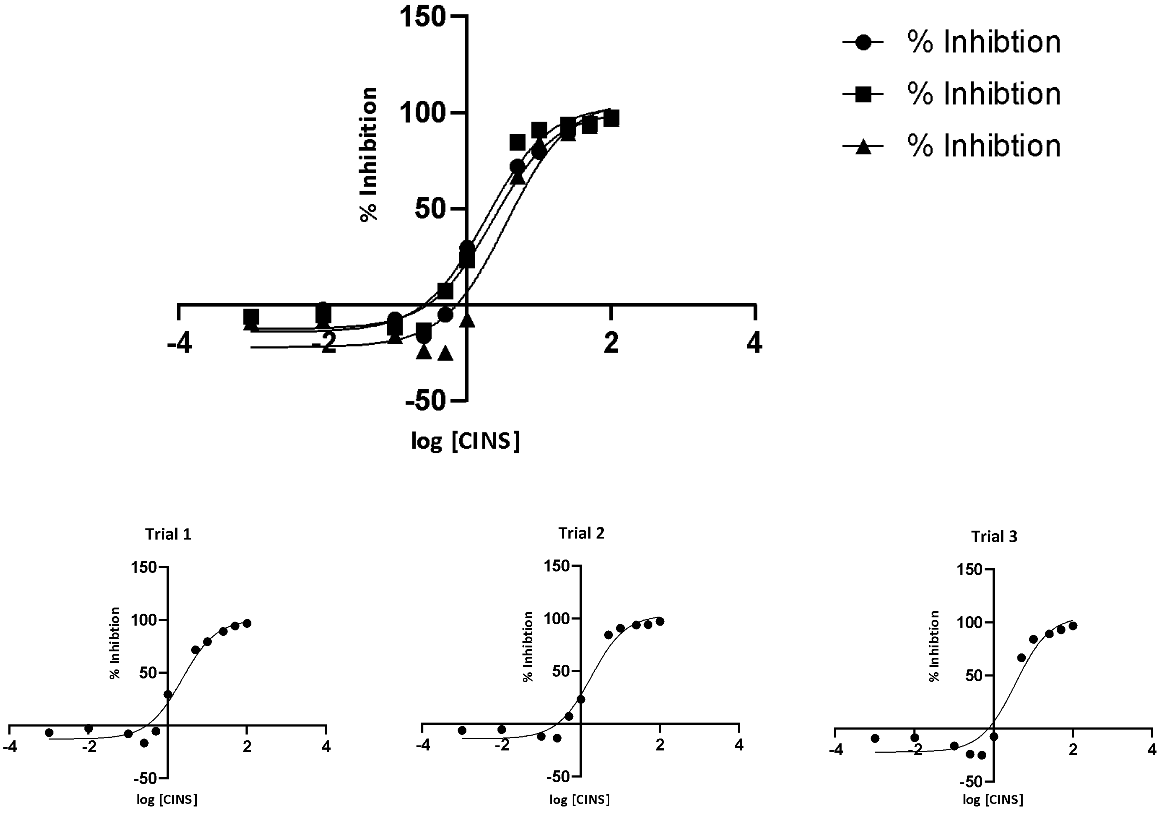

Supplementary Figure 16. Dose-response curves of CINS against LOX.

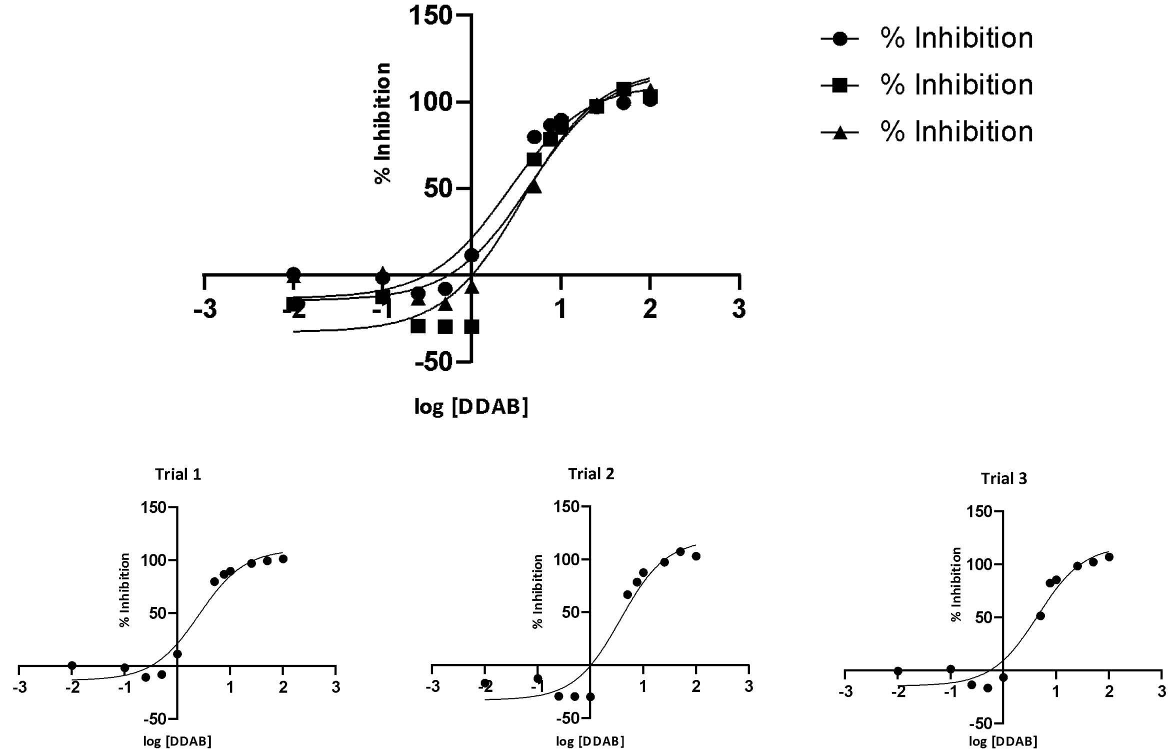

Supplementary Figure 17. Dose-response curves of DDAB against LOX.

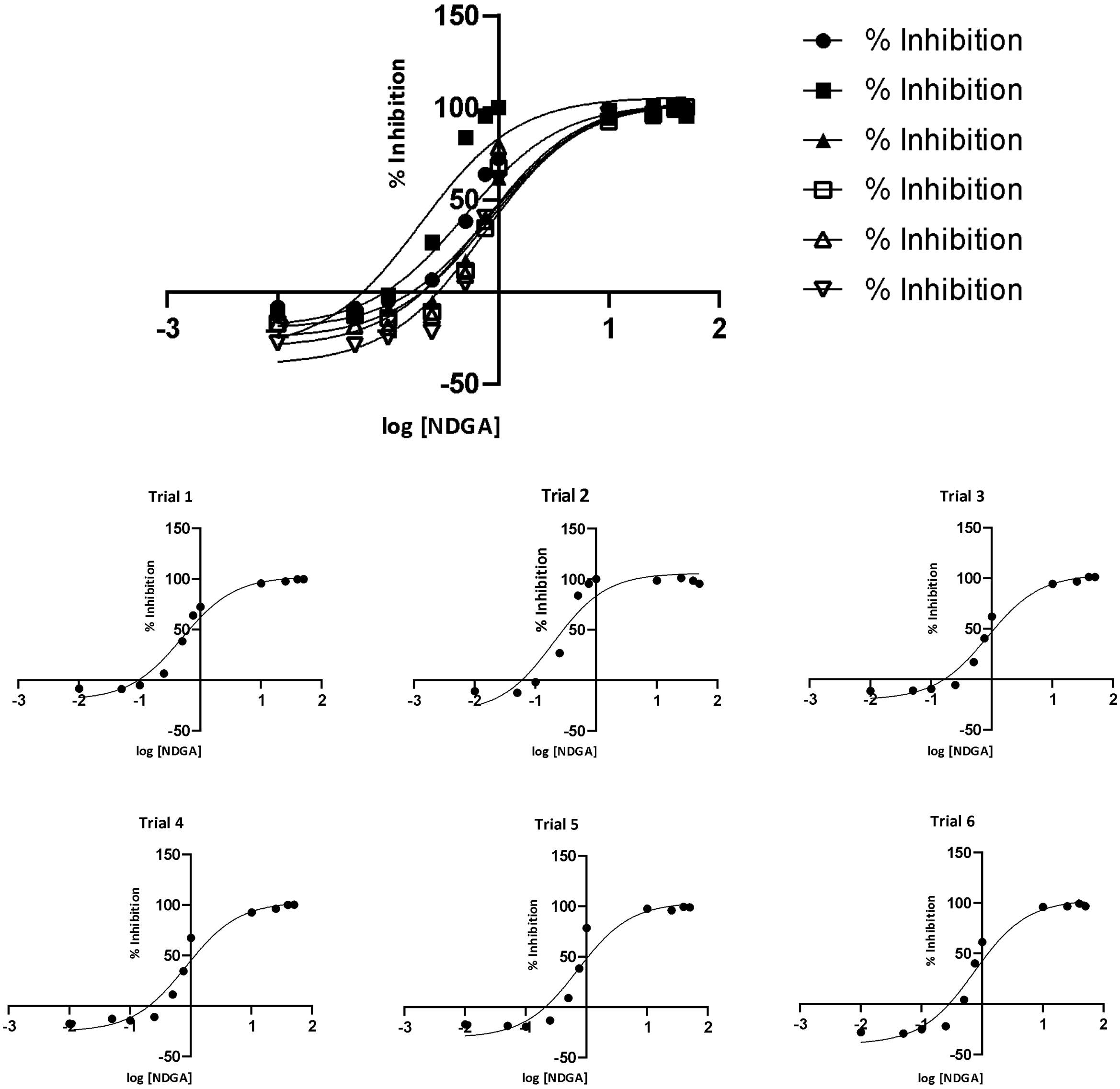

Supplementary Figure 18. Dose-response curves of NDGA against LOX.

1.2 Supplementary Tables

Supplementary Table 1. Effective concentrations of CINS, DDAB, and acarbose for the α-glucosidase inhibition assay.

[CINS], μg/mL	[DDAB], μg/mL	[acarbose], μg/mL
0.001	0.0001	15
0.01	0.001	30
0.10	0.01	50
0.25	0.10	100
0.50	0.25	150
1.00	0.40	200
2.00	0.50	300
5.00	1.00	500
10.00	2.00	750
25.00	10.00	1500
75.00	25.00	3000
-	-	4500

Supplementary Table 2. Effective concentrations of CINS, DDAB, and nordihydroguaiaretic acid (NDGA) for the 15-lipoxygenase-1 inhibition assay.

[CINS], μg/mL	[DDAB], μg/mL	[NDGA], μg/mL
0.001	0.01	0.01
0.01	0.10	0.05
0.10	0.25	0.10
0.25	0.50	0.25
0.50	1.00	0.50
1.00	5.00	0.75
5.00	7.50	1.00
10.00	10.00	10.00
25.00	25.00	25.00
50.00	50.00	40.00
100.00	100.00	50.00

Supplementary Table 3. Statistical analysis of the IC_{50} calculation for CINS against AGLUC.

log(inhibitor) vs. response (three parameters)	Trial 1	Trial 2	Trial 3
Best-fit values			
Bottom	106.3	105.3	106.2
Top	-17.82	-0.9047	-9.288
LogIC50	-0.5039	-0.7069	-0.617
IC50	0.3134	0.1964	0.2415
Span	-124.1	-106.2	-115.5
95%CI (profile likelihood)			
Bottom	90.15 to 123.3	94.60 to 116.3	93.56 to 119.4
Top	-41.07 to 4.419	-17.98 to 15.55	-28.62 to 9.324
LogIC50	-0.8408 to -.01646	-0.9976 to -0.4284	-0.9127 to -0.3274
IC50	0.1443 to 0.6845	0.1006 to 0.3729	0.1223 to 0.4705
Goodness of Fit			
Degrees of Freedom	8	8	8
R squared	0.9323	0.9526	0.9468
Sum of Squares	1658	802.5	1092
Sy.x	14.39	10.02	11.68
Number of points			
# of X values	11	11	11
# of Y values analyzed	11	11	11
IC50 Mean	0.25043		
IC50 Std Dev	0.059		

Supplementary Table 4. Statistical analysis of the IC_{50} calculation for DDAB against AGLUC.

log(inhibitor) vs. response (three parameters)	Trial 1	Trial 2	Trial 3
Best-fit values			
Bottom	107.1	109.6	108.5
Top	-12.43	-14.2	-10.22
LogIC50	-0.5557	-0.682	-0.5909
IC50	0.2782	0.208	0.2565
Span	-119.5	-123.8	-118.7
95%CI (profile likelihood)			
Bottom	95.61 to 119.1	91.19 to 129.4	94.81 to 122.9
Top	-23.02 to -2.008	-33.08 to 4.284	-23.34 to 2.674
LogIC50	-0.7556 to -0.3679	-1.019 to -0.3831	-0.8286 to -0.3684
IC50	0.1755 to 0.4287	0.09573 to 0.4139	0.1484 to 0.4281
Goodness of Fit			
Degrees of Freedom	8	8	8
R squared	0.9771	0.9378	0.9647
Sum of Squares	496	1549	769.8
Sy.x	7.874	13.91	9.809
Number of points			
# of X values	11	11	11
# of Y values analyzed	11	11	11
IC50 Mean	0.24757		
IC50 Std Dev	0.035		

Supplementary Table 5. Statistical analysis of the IC_{50} calculation for acarbose against AGLUC.

log(inhibitor) vs. response (three parameters)	Trial 1	Trial 2	Trial 3	Trial 4	Trial 5	Trial 6
Best-fit values						
Bottom	99.12	99.7	98.49	98.72	99.41	98.87
Top	-1.837	10.53	-5.809	-7.329	-5.78	-5.368
LogIC50	2.143	2.141	2.015	2.1	2.148	2.114
IC50	139	138.2	103.4	125.9	140.5	130.1
Span	-101	-89.17	-104.3	-106	-105.2	-104.2
95%CI (profile likelihood)						
Bottom	96.49 to 101.8	95.38 to 104.3	95.74 to 101.3	93.86 to 103.8	95.74 to 103.2	97.35 to 100.4
Top	-6.450 to 2.475	2.647 to 17.50	-12.01 to -0.1837	-16.97 to 1.090	-12.23 to 0.1373	-8.111 to -2.732
LogIC50	2.071 to 2.214	2.006 to 2.273	1.929 to 2.098	1.964 to 2.232	2.052 to 2.243	2.073 to 2.155
IC50	117.6 to 163.9	101.3 to 187.5	85.01 to 125.2	92.09 to 170.7	112.6 to 174.8	118.3 to 142.9
Goodness of Fit						
Degrees of Freedom	9	9	9	9	9	9
R squared	0.9974	0.9906	0.9967	0.9913	0.9953	0.9991
Sum of Squares	27.83	78.96	34.71	101.4	55.13	9.536
Sy.x	1.759	2.962	1.964	3.356	2.475	1.029
Number of points						
# of X	12	12	12	12	12	12

values						
# of Y values analyzed	12	12	12	12	12	12
IC50 Mean	129.52					
IC50 Std Dev	14.01					
# of values	6					
Significance level	0.05 (two-sided)					
Critical value of Z	1.8871466793					
Trial #	IC50	Z	Significant outlier?			
1	139.0	0.677				
2	138.2	0.620				
3	103.4	1.864	Furthest from the rest, but not a significant outlier (P>0.05).			
4	125.9	0.258				
5	140.5	0.784				
6	130.1	0.042				

Supplementary Table 6. Statistical analysis of the IC_{50} calculation for CINS against LOX.

log(inhibitor) vs. response (three parameters)	Trial 1	Trial 2	Trial 3
Best-fit values			
Bottom	100.7	103.6	106.4
Top	-12.54	-14.19	-22.13
LogIC50	0.3666	0.2692	0.5479
IC50	2.326	1.859	3.531
Span	-113.2	-117.8	-128.5
95%CI (profile likelihood)			
Bottom	88.09 to 114.8	92.15 to 115.9	86.77 to 129.1
Top	-23.76 to -1.856	-25.20 to -3.648	-37.19 to -7.678
LogIC50	0.09512 to 0.6564	0.03381 to 0.5183	0.2177 to 0.8884
IC50	1.245 to 4.533	1.081 to 3.298	1.651 to 7.735
Goodness of Fit			
Degrees of Freedom	8	8	8
R squared	0.9722	0.9763	0.9526
Sum of Squares	630.9	586.5	1362
Sy.x	8.88	8.562	13.05
Number of points			
# of X values	11	11	11
# of Y values analyzed	11	11	11
IC50 Mean	2.572		
IC50 Std Dev	0.8627		

Supplementary Table 7. Statistical analysis of the IC_{50} calculation for DDAB against LOX.

log(inhibitor) vs. response (three parameters)	Trial 1	Trial 2	Trial 3
Best-fit values			
Bottom	110.3	119	117.8
Top	-13.29	-32.83	-14.6
LogIC50	0.4101	0.5673	0.6519
IC50	2.571	3.692	4.486
Span	-123.6	-151.9	-132.4
95%CI (profile likelihood)			
Bottom	95.64 to 126.8	94.85 to 147.5	97.88 to 141.1
Top	-28.28 to 0.7331	-54.03 to -12.85	-30.32 to 0.3843
LogIC50	0.1270 to 0.6884	0.2206 to 0.9008	0.3373 to 0.9518
IC50	1.340 to 4.880	1.662 to 7.958	2.174 to 8.950
Goodness of Fit			
Degrees of Freedom	8	8	8
R squared	0.9641	0.9438	0.955
Sum of Squares	887.8	2070	1209
Sy.x	10.53	16.09	12.3
Number of points			
# of X values	11	11	11
# of Y values analyzed	11	11	11
IC50 Mean	3.583		
IC50 Std Dev	0.9621		

Supplementary Table 8. Statistical analysis of the IC_{50} calculation for NDGA against LOX.

log(inhibitor) vs. response (three parameters)	Trial 1	Trial 2	Trial 3	Trial 4	Trial 5	Trial 6
Best-fit values						
Bottom	102.5	106.2	104	103.3	104	103.3
Top	-19.43	-31.68	-20.04	-25.15	-30.07	-39.64
LogIC50	-0.3013	-0.701	-0.06799	-0.07787	-0.1428	-0.1293
IC50	0.4997	0.1991	0.8551	0.8359	0.7198	0.7425
Span	-121.9	-137.8	-124.1	-128.4	-134.1	-142.9
95%CI (profile likelihood)						
Bottom	92.46 to 112.7	89.50 to 123.4	93.39 to 115.0	89.54 to 117.5	86.11 to 122.5	88.82 to 118.1
Top	-34.19 to -5.626	-67.58 to -1.277	-33.41 to -7.362	-42.65 to -8.764	-53.93 to -8.179	-58.58 to -21.91
LogIC50	-0.5040 to -0.09315	-1.055 to -0.3631	-0.2706 to 0.1572	-0.3287 to 0.2071	-0.4520 to 0.2058	-0.3663 to 0.1311
IC50	0.3133 to 0.8070	0.08803 to 0.4334	0.5363 to 1.436	0.4691 to 1.611	0.3532 to 1.606	0.4302 to 1.353
Goodness of Fit						
Degrees of Freedom	8	8	8	8	8	8
R squared	0.9743	0.9204	0.9766	0.9634	0.9409	0.966
Sum of Squares	562	1952	569.9	963.9	1705	1091
Sy.x	8.382	15.62	8.44	10.98	14.6	11.68
Number of points						
# of X	11	11	11	11	11	11

values						
# of Y values analyzed	11	11	11	11	11	11
IC50 Mean	0.6420					
IC50 Std Dev	0.2512					
